# Optimizing surveillance in Lynch syndrome: lesion detection and comparative performance of different colonoscopy modalities—a systematic review and network meta-analysis

**DOI:** 10.1007/s00384-025-04970-2

**Published:** 2025-08-12

**Authors:** George Hanen, Hazem E. Mohammed, Mohamed Nasser, Mohamed E. Haseeb, Hatem Yaser, Shehab Yaser, Salma Allam

**Affiliations:** 1 School of Medicine, Minia University, Minia, Egypt; 2 Faculty of Medicine, Assiut University, Assiut, Egypt; 3 Medical Research Group of Egypt (MRGE), Negida Academy, Cairo, Egypt; 4 Faculty of Medicine, Galala University, Suez, Egypt

**Keywords:** Lynch syndrome, Hereditary nonpolyposis colorectal cancer, Colonoscopy, Systematic review, Network meta-analysis

## Abstract

**Purpose:**

Lynch syndrome patients are at a high risk for developing colorectal cancer; thus, optimal surveillance strategies are required. Although colonoscopic imaging methods differ in diagnostic performance, direct comparisons in this population are not very common. We aimed to evaluate and compare the detection capabilities of white-light endoscopy (WLE), chromoendoscopy, virtual chromoendoscopy (NBI: narrow band imaging, LCI: linked color imaging, I-SCAN), and AI-assisted colonoscopy in detecting neoplastic and non-neoplastic lesions in individuals diagnosed with Lynch syndrome.

**Methods:**

Up until March 2025, PubMed, WOS, and Scopus were searched. Relevant studies included observational or interventional designs that contrasted various forms of colonoscopy in adults with Lynch syndrome. The primary outcomes were the lesion detection rate and number of lesions per colonoscopy. Secondary outcomes included total procedure time and withdrawal time. Credibility of the evidence was assessed employing CINeMA.

**Results:**

Nine studies were included. LCI and chromoendoscopy demonstrated a significantly higher neoplastic lesion detection rate compared to WLE (RD 0.11, 95% CI [0.01, 0.21], *P* = 0.03) and (RD 0.07, 95% CI [0.01, 0.14], *P* = 0.03), respectively, and LCI significantly detected more lesions per procedure (MD = 0.23, 95% CI 0.01–0.45, *P* = 0.04). Chromoendoscopy was better at marking the non-neoplastic lesions (RD 0.16, 95% CI [0.05, 0.27], *P* = 0.005) but had the longest procedure and withdrawal times. AI-assisted, as well as virtual ones, were better than WLE but were not as effective as LCI or chromoendoscopy.

**Conclusion:**

In terms of efficiency, LCI and chromoendoscopy improved WLE in detecting neoplastic lesions in Lynch syndrome. Chromoendoscopy remains valuable for non-neoplastic detection, but procedural time is a major drawback. AI-assisted technologies are promising, which require additional investigation.

**Supplementary Information:**

The online version contains supplementary material available at https://doi.org/10.1007/s00384-025-04970-2.

## Introduction

Lynch syndrome, an autosomal dominant disorder also known as hereditary non-polyposis colorectal cancer [1–3], is one of the most common hereditary cancer syndromes [4]. Lynch patients may also have other cancers, such as brain, stomach, ovarian, or endometrial cancer [5–7].

Lynch syndrome is caused by germline mutations in DNA mismatch repair (MMR) genes, including MLH1, MSH2, MSH6, PMS2, and EpCAM [1, 2, 7], that result in a condition known as microsatellite instability, also referred to as the mutational signature of tumors [8–10]. Normally, during DNA replication, mismatch repair errors, such as base mismatch or insertion loop deletion, occur, and the MMR system corrects these errors. In Lynch syndrome, this system malfunctions, causing adenomas and cancer to develop, but colorectal cancer (CRC) is the most common type to occur [11–13].

Lynch syndrome is responsible for 2–4% of all cases of colorectal cancer [2, 14]. The lifetime risk of colorectal cancer in patients with Lynch syndrome ranges from 20 to 80%. The main precursor to colorectal cancer in Lynch syndrome is adenoma, which usually progresses more rapidly to cancer because it usually has a villous component or high-grade dysplasia [7]. Colonoscopy is the gold standard for screening in Lynch syndrome and for adenoma detection. As a result, pancolonoscopies should be performed every 1 to 2 years according to current European and North American standards [15, 16].

While the colonoscopy indicators, such as bowel preparation and endoscopist skills, may affect the potential colonoscopy to prevent CRC development, the type of colonoscopy may play a crucial role in increasing the adenoma detection rate, as there is a significant number of adenomas missed, with rates ranging from 12 to 62%, and also between 2 and 6% of larger polyps or carcinomas can be missed [15, 17–19].

There are different kinds of colonoscopy, like standard white-light endoscopy (WLE), dye-based chromoendoscopy (CE) that uses indigo carmine dye, narrow-band imaging colonoscopy (NBI), which utilizes specific wavelengths of light to detect mucosal and submucosal abnormalities with better contrast, and linked color imaging (LCI) endoscopy, which is based on the same principle as NBI, however, with additional digital enhancement of image colors. Other methods include virtual colonoscopy, which is actually an enhanced CT or MR colonography, and AI-assisted detection systems, or computer-aided detection systems (CADe), but there is a lot of debate about which one detects the most adenomas and misses the fewest. Rivero-Sanchez et al. reported that dye-based chromoendoscopy is a difficult, permanent, and time-consuming process. Rivero-Sanchez et al. reported that dye-based chromoendoscopy is a laborious, irreversible, and time-consuming procedure [7], while Huneburg et al. discovered that chromocolonoscopy is superior to NBI [4]. Moreover, Ortiz et al. discovered that WLE has a higher adenoma detection rate than CADe [1], and Montale et al. reported that CE is not superior to high-resolution WLE [2]. As a result, we conducted this systematic review and network meta-analysis to provide clear and transparent comparisons between all these modalities and describe the best type with the highest detection rates for neoplastic and non-neoplastic colonic lesions. Furthermore, we assessed the credibility of evidence employing the Confidence in Network Meta-Analysis (CINeMA) web application.

## Methods

This systematic review and network meta-analysis was conducted in concordance with the Preferred Reporting Items for Systematic Review and Meta-analysis (PRISMA) statement and the PRISMA extension statement for reporting of systematic reviews incorporating network meta-analyses of health care interventions [20]. A protocol was registered on the PROSPERO database (CRD420251012538) [21].

### Eligibility criteria

The included studies should meet the following criteria:

1. Controlled studies, either interventional or observational
2. Studies comparing any type of colonoscopy with any other type (e.g., WLE vs. chromoendoscopy)
3. Studies including adult patients (≥ 18 years) diagnosed with Lynch syndrome either by evidence of (likely) pathogenic germline mutation in mismatch repair (MMR) genes (MLH1, MSH2, MSH6, PMS2) or the Epcam gene or by fulfilling the strict Amsterdam criteria [22]
4. Full articles in English language only

Case reports or series, conference abstracts, and studies not written in English were excluded. Moreover, any studies that excised the colonic lesions after performing the first colonoscopy were excluded.

### Search strategy

PubMed, Scopus, and WOS databases were extensively searched from the time of inception until March 2025 using the following examples of keywords and Medical Science Heading (MeSH) terms: “Lynch Syndrome,” “Hereditary nonpolyposis colorectal cancer,” “Colonoscopy,” “White-light endoscopy,” “Chromoendoscopy,” “Virtual chromoendoscopy,” “Narrow band imaging (NBI),” “Linked color imaging (LCI),” “I-SCAN,” and “AI-assisted colonoscopy.” The full search strategy is demonstrated in Supplementary Table 1. Duplicated studies were removed using Endnote software [22].

### Study selection and data extraction

Two independent authors conducted title and abstract screening followed by full-text screening of the retrieved studies using Rayaan online software [23]. A third author was involved in resolving conflicts in each screening stage. Data extraction was conducted using an online Excel sheet for easy accessibility. Four authors, divided into two groups, conducted data extraction independently, and then, any conflicts were resolved by discussion or by an independent third party. The online data extraction sheet included study characteristics, baseline data for studies’ populations, and outcome measures data. Study characteristics included study name and year, groups of comparison, design, sample size, location and duration of the study, and key findings. Population baseline data included age, gender, extent of colonoscopy, and time since last colonoscopy. Primary outcome measures data included:

1. Lesion detection rate that is defined as the proportion of patients with at least one identified lesion. We extracted lesion detection rates for non-neoplastic and neoplastic lesions.
2. Number of lesions per colonoscopy that is defined as the total number of lesions detected divided by the total number of colonoscopies performed. We extracted the number of lesions per colonoscopy for non-neoplastic and neoplastic lesions [24, 25].

Secondary outcome measures included:

1. Total procedure time is defined as the time from starting endoscopy insertion to completing withdrawal of the endoscope.
2. Withdrawal time is defined as the time from intubation till withdrawal of endoscopy from the anal verge; it corresponds to the time spent on inspection of the bowel [26].

### Quality assessment

The quality of included studies was assessed using the revised Cochrane risk-of-bias tool for randomized trials (ROB2) that included five domains: randomization process, deviations from the intended interventions, risk of bias due to missing outcome data, measurement of the outcome, and selection of the reported result [27]. Moreover, we employed the Newcastle–Ottawa Scale (NOS) for observational cohort studies that assesses selection, comparability of cohorts, and outcome assessment [28]. These assessments informed the “within-study bias” domain in the CINeMA framework, where comparisons involving higher-risk studies (e.g., the cohort study) were downgraded accordingly.

Two authors conducted quality assessment independently, and any conflicts were resolved either by discussion or by a third author. Studies were categorized as low risk, some concerns, or high risk of bias based on ROB2 criteria and were categorized as poor, fair, or good quality based on NOS criteria.

### Statistical analysis

To carry out the network meta-analysis, we used RStudio [29] and followed a frequentist framework with a random-effects model using the “netmeta” package [30]. This method allowed us to compare several treatments by combining both direct evidence (from head-to-head trials) and indirect evidence (from studies sharing a common comparator). For outcomes reported on a continuous scale, we expressed effect sizes as mean differences (MD), while for binary outcomes, we used risk difference (RD), both accompanied by 95% confidence intervals. Heterogeneity was assessed using both Cochran’s *Q* test and the I^2^ statistic. A significant *Q* test (*P* < 0.10) and an *I*^2^ value ≥ 50% were considered indicative of substantial heterogeneity [31].

We evaluated the relative performance of each treatment using the Surface Under the Cumulative Ranking Curve (SUCRA), where higher SUCRA values suggest a greater likelihood of being the most effective option. To visualize the network of treatment comparisons, we created network plots, and we illustrated individual treatment effects using forest plots. Additionally, league tables were used to summarize and rank all interventions, making it easier to compare their overall performance. For each outcome, only studies reporting the relevant data were included in that specific network analysis.

### Certainty of evidence

We assessed the credibility of each active control comparison using CINeMA [32, 33], an online tool adapted from GRADE for network meta-analyses by the Cochrane Comparing Multiple Interventions Methods Group. Each comparison was evaluated across six domains: within-study bias, reporting bias, indirectness, imprecision, heterogeneity, and incoherence. Confidence ratings started at high and were downgraded based on concerns within each domain.

Within-study bias was downgraded when the majority of contributing trials had an unclear or high risk of bias. Reporting bias was suspected when asymmetry was observed in funnel plots; however, most comparisons included fewer than 10 studies, limiting the reliability of funnel plot interpretation [34]. Indirectness was considered when study populations lacked generalizability (e.g., with respect to age, sex, or comorbidities); one such concern led to a “some concerns” rating, while two or more indicated “major concerns.”

Imprecision was judged using a pre-specified clinical equivalence margin of ± 10% absolute risk difference. Confidence intervals crossing one margin were rated as having “some concerns,” while those crossing both margins were rated as “major concerns.” Heterogeneity concerns were raised when confidence and prediction intervals led to different conclusions regarding clinical importance. Incoherence was assessed using both global and local methods (e.g., node-splitting), with downgrading applied when direct and indirect comparisons yielded substantially different estimates.

## Results

### Literature search

**Fig. 1:**
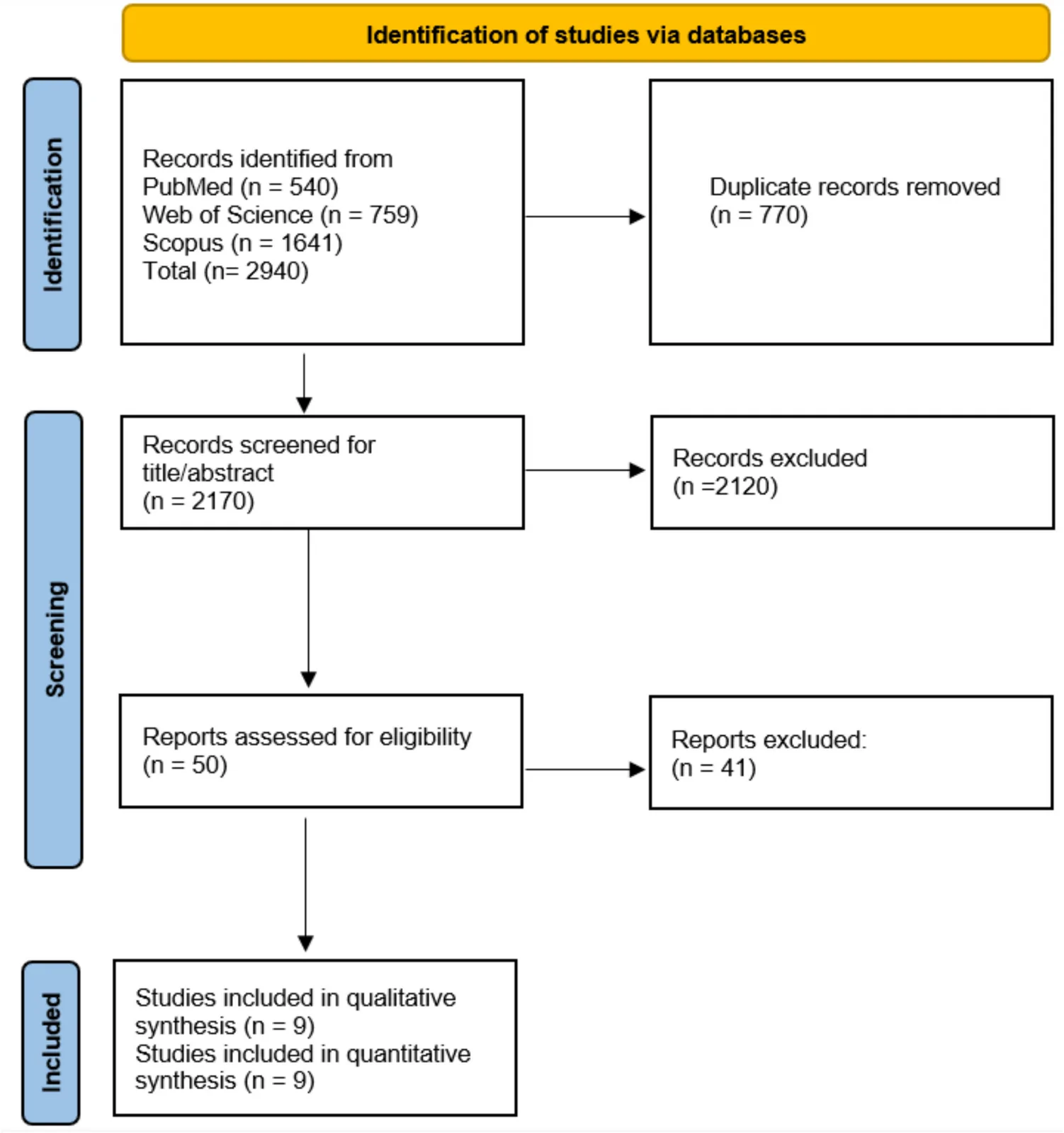
PRISMA flow diagram

Our search strategy initially retrieved 2940 records. After removing 770 duplicates, 2170 unique records remained. These were carefully screened based on their titles and abstracts, which narrowed the pool down to 50 articles selected for full-text review. Following this detailed assessment, nine studies met the eligibility criteria and were included in both the qualitative and quantitative analyses. A detailed overview of the selection process is illustrated in the PRISMA flow diagram, as shown in Fig. 1.

### Study and population characteristics

Our network meta-analysis encompassed nine studies with a combination of randomized controlled trials (RCTs) [1, 3, 4, 7, 15, 17, 35, 36] and a single retrospective cohort study [2], where patient populations were recruited from various centers in Europe and the USA. Sample sizes in the studies differed, ranging from a minimum of 13 participants to almost 700 in certain comparisons. The distribution of male participants ranged between 15 and 53%. The mean age of the patients ranged from the early 40 s to late 50 s, with variation based on the specific study population. Time since the last prior endoscopic exam was reported in most studies, with average intervals ranging from 12 to more than 60 months. Although some trials were directed to the proximal colon, the majority of studies involved examination over the whole colon (pancolic extent). Studies were carried out in various academic and community-based centers, reflecting the diversity of clinical environments. Studies and population baseline characteristics are represented in Table 1. Furthermore, the reported detection rates in all the individual included studies for neoplastic and non-neoplastic lesions are summarized and reported in Supplementary Tables 4 and 5.

**Table 1:** Studies and population baseline characteristics of the included studies

Study ID	Arms	Sample size	Male, *n* (%)	Age (years), mean (SD)	Time since last endoscopy mean in months, mean (SD)	Study location and duration	Extent of endoscopy	Key findings
Hüneburg et al. [4] (RCT)	WLE	47	NA	43.1 (10.9)	12.3 (7.6)	The Department of Internal Medicine of the University of Bonn	Proximal colon	Chromocolonoscopy surpassed NBI and standard white-light colonoscopy in the detection of hyperplastic lesions, especially adenomatous lesions
	Chromoendoscopy	47						
	NBI	62		46.9 (11.3)	12.1 (5.2)			
	Chromoendoscopy	62						
Haanstra et al. [35] (RCT)	WLE	116	46 (39)	46 (11)	NA	6 centers in Netherlands. Patients were recruited between July 2008 to June 2014	Proximal colon	Neoplasm detection rate was nearly the same in both groups. Proximal colon detection rate was slightly more for WLE versus that for CE. Total procedural time was 9 min longer in CE group
	Chromoendoscopy	115	49 (43)	46 (12)				
Houwen et al. [17] (RCT)	WLE	172	67 (39)	47.32 (13.8)	18.33 (8.22)	8 centers in Belgium, Italy, the Netherlands, Poland, Spain, and the UK. Between January 2018 and March 2020	Pancolic	LCI significantly improved detection rate of adenomas compared to HD-WLE
	LCI	160	74 (46)	49.6 (14.4)	18 (9.73)			
Hüneburg et al. [3] (RCT)	WLE	46	21 (45.7)	46.3 (11.8)	17.0 (5.6)	The National Center for Hereditary Tumor Syndromes in Bonn, Germany. Between December 2021 and December 2022	Pancolic	HD-WLE did not significantly improve detection rate of polyps or adenomas compared to CADEYE
	AI-assisted system	50	20 (40)	50.3 (11.9)	16.5 (5.3)			
Montale et al. [2] (Retrospective cohort)	WLE	29	11 (37.9)	44.4 (10.8)	62.6 (41.8)	The Endoscopic Unit of the S.Orsola-Malpighi Hospital, University of Bologna, Italy. From 2007 to 2019	Pancolic	HD-WLE did not significantly improve detection rate of polyps or adenomas compared to CE
	Chromoendoscopy	13	2 (15)	40.4 (9.7)	38.0 (25.0)			
Ortiz et al. [1] (RCT)	WLE	216	87 (40)	50.14 (14.47)	NA	11 academic centers and 6 community centers in Belgium, Germany, Italy, and Spain with 30 endoscopists. Between September 13, 2021, and April 6, 2023	Pancolic	CADe GI Genius did not significantly improve detection rate of polyps or adenomas compared to HD-WLE
	AI-assisted system	214	87 (41)	47.78 (13.97)	NA			
Rivero-Sánchez et al. [7] (RCT)	WLE	128	57 (44.53)	47.6 (13.6)	16.7 (7)	14 Spanish centers by 24 participating endoscopists from July 2016 to January 2018	Pancolic	HD-WLE significantly improved polyp detection compared to CE, but its impact on adenoma detection was not significant
	Chromoendoscopy	128	46 (35.94)	46.7 (14.3)	17.8 (13.5)			
Wei et al. [36] (RCT)	WLE	382	198 (51.2)	57.6 (7.9)	NA	4 community-based endoscopy centers located in California, Connecticut, Maryland, and New Jersey. Between September 28, 2020, and September 24, 2021	Pancolic	CADe EndoVigilant did not significantly improve detection rate of adenomas compared to WLE
	AI-assisted system	387	194 (50.8)	58.0 (7.9)	NA			
Bisschops et al. [15] (RCT)	WLE	31	14 (45.16)	46 (12.27)	NA	The Department of Gastroenterology/Endoscopy in the University Hospital Leuven. Between November 2010 and April 2012	Pancolic	I-SCAN improved detection rate of polyps and adenomas compared to HD-WLE
	I-SCAN	30	16 (53.33)	43 (10.71)	NA			

WLE, white-light endoscopy; NBI, narrow band imaging; CE, chromoendoscopy; LCI, linked color imaging; HD-WLE, high-definition white-light endoscopy; AI, artificial intelligence; NA, not available

### Quality assessment

The risk of bias assessment for RCTs was conducted using the ROB 2 tool, as shown in Supplementary Fig. 1. Most studies demonstrated a low risk of bias across some domains. However, there were some concerns for certain studies regarding the deviations from intended interventions (D2) and the measurement of the outcomes (D4). For the cohort study included [2], quality was evaluated using the Newcastle–Ottawa Scale (NOS) and was determined to be 7/9, indicating good methodological quality, as represented in Supplementary Table 2. Overall, the included studies were of moderate to high quality, in favor of the strength of the network meta-analysis findings.

### Network meta-analysis

#### Primary outcomes

##### Lesion detection rate

**Fig. 2:**
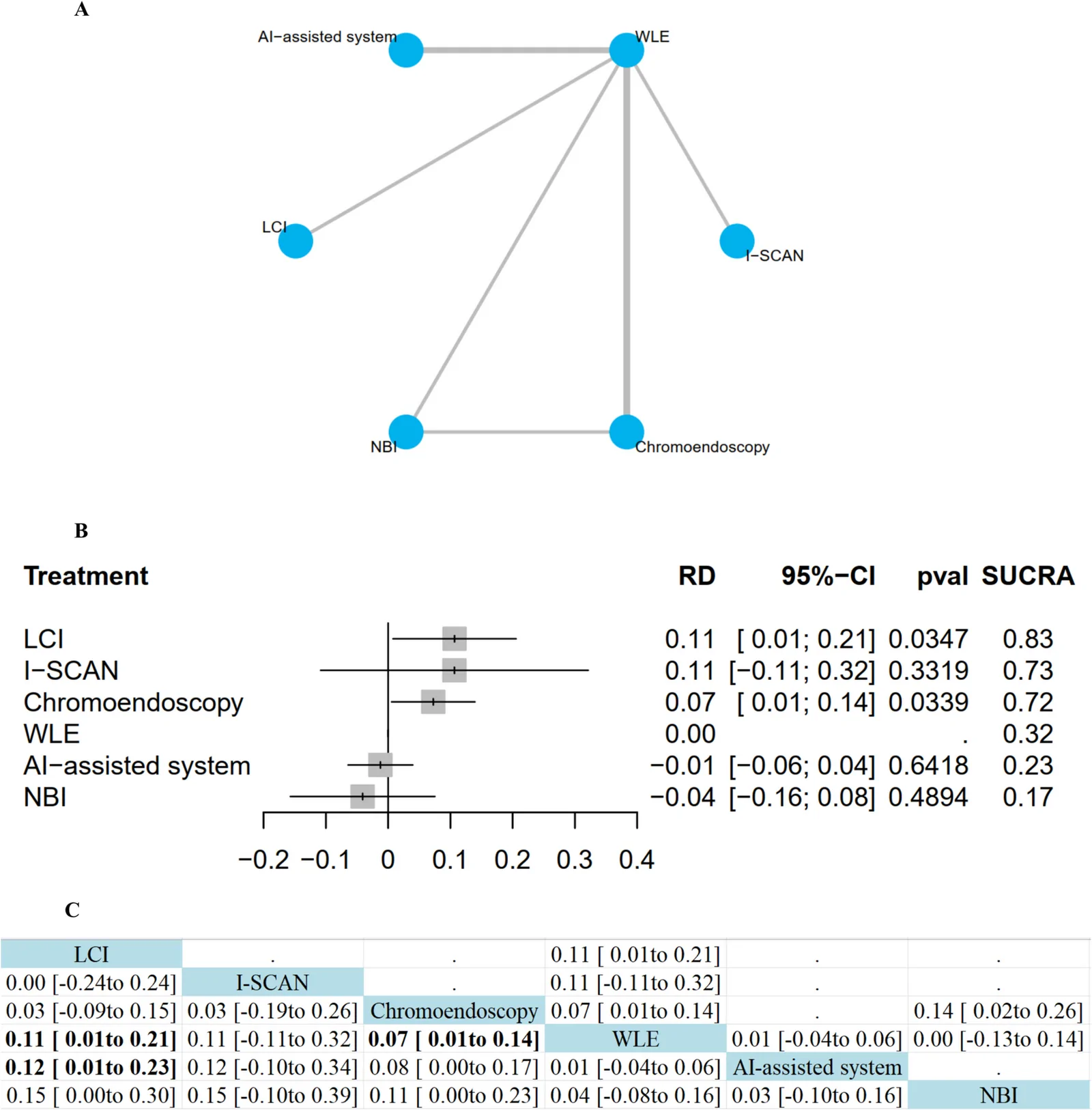
Lesion detection rate for neoplastic lesions: **A** network plot, **B** forest plot, and **C** league table

Regarding neoplastic lesions, nine studies reported the lesion detection rate. The network plot for the neoplastic lesions’ detection rate is presented in Fig. 1A. Only LCI and chromoendoscopy showed statistically significant detection rates of neoplastic lesions compared to WLE (RD 0.11, 95% CI [0.01, 0.21], *P* = 0.03) and (RD 0.07, 95% CI [0.01, 0.14], *P* = 0.03), as shown in Fig. 2B. Besides, LCI demonstrated a significantly higher detection rate compared to the AI-assisted system (RD 0.12, 95% CI [0.01, 0.23], as shown in Fig. 2C. No significant difference existed between the other modalities. According to surface under the cumulative ranking curve (SUCRA) rankings, LCI achieved the highest ranking (SUCRA = 83%), followed by I-SCAN (SUCRA = 73%), chromoendoscopy (SUCRA = 72%), WLE (SUCRA = 32%), AI-assisted system (SUCRA = 23%), and NBI (SUCRA = 17%). Besides, the analysis revealed no substantial heterogeneity or inconsistency (Cochran’s *Q* = 3.01, *P* = 0.6992, with *I*^2^ = 0%).

**Fig. 3:**
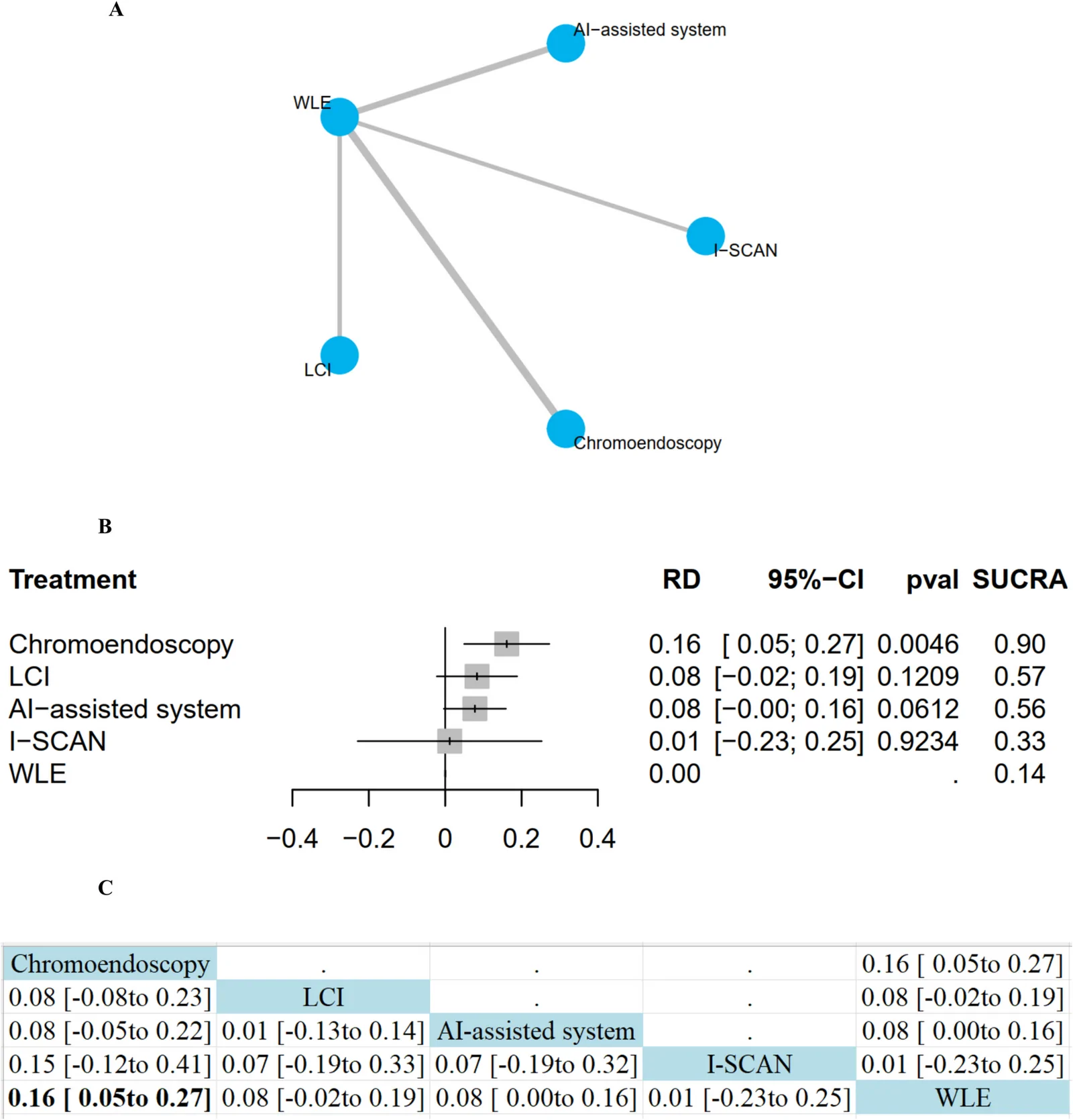
Lesion detection rate for non-neoplastic lesions: **A** network plot, **B** forest plot, and **C** league table

With respect to non-neoplastic lesions, six studies were involved in the analysis. The network plot for non-neoplastic lesions detection rate is presented in Fig. 3A. Only chromoendoscopy showed a statistically significant detection rate of non-neoplastic lesions compared to WLE (RD 0.16, 95% CI [0.05, 0.27], *P* = 0.005, as shown in Fig. 3B). No significant difference existed between any of the other arms. According to SUCRA rankings, chromoendoscopy ranked first (SUCRA = 90%), while WLE ranked last (SUCRA = 14%). Besides, the analysis revealed no substantial heterogeneity or inconsistency (Cochran’s *Q* = 0.36, *P* = 0.8339, with *I*^2^ = 0%).

Regarding morphology in the detection of non-neoplastic lesions, although LCI ranked first (SUCRA = 68%) in the detection of hyperplastic polyps, no significant difference existed when compared to WLE or between any of the other modalities (Supplementary Fig. 2). The analysis revealed no substantial heterogeneity or inconsistency (Cochran’s *Q* = 0.03, *P* = 0.8739, with *I*^2^ = 0%).

For sessile polyps, the AI-assisted system achieved the first rank (SUCRA = 84%); however, no significant difference existed when compared to WLE or between any of the other modalities (Supplementary Fig. 3). No substantial heterogeneity or inconsistency was detected (Cochran’s *Q* = 0.05, *P* = 0.8871, with *I*^2^ = 0%).

##### Number of lesions per colonoscopy

**Fig. 4:**
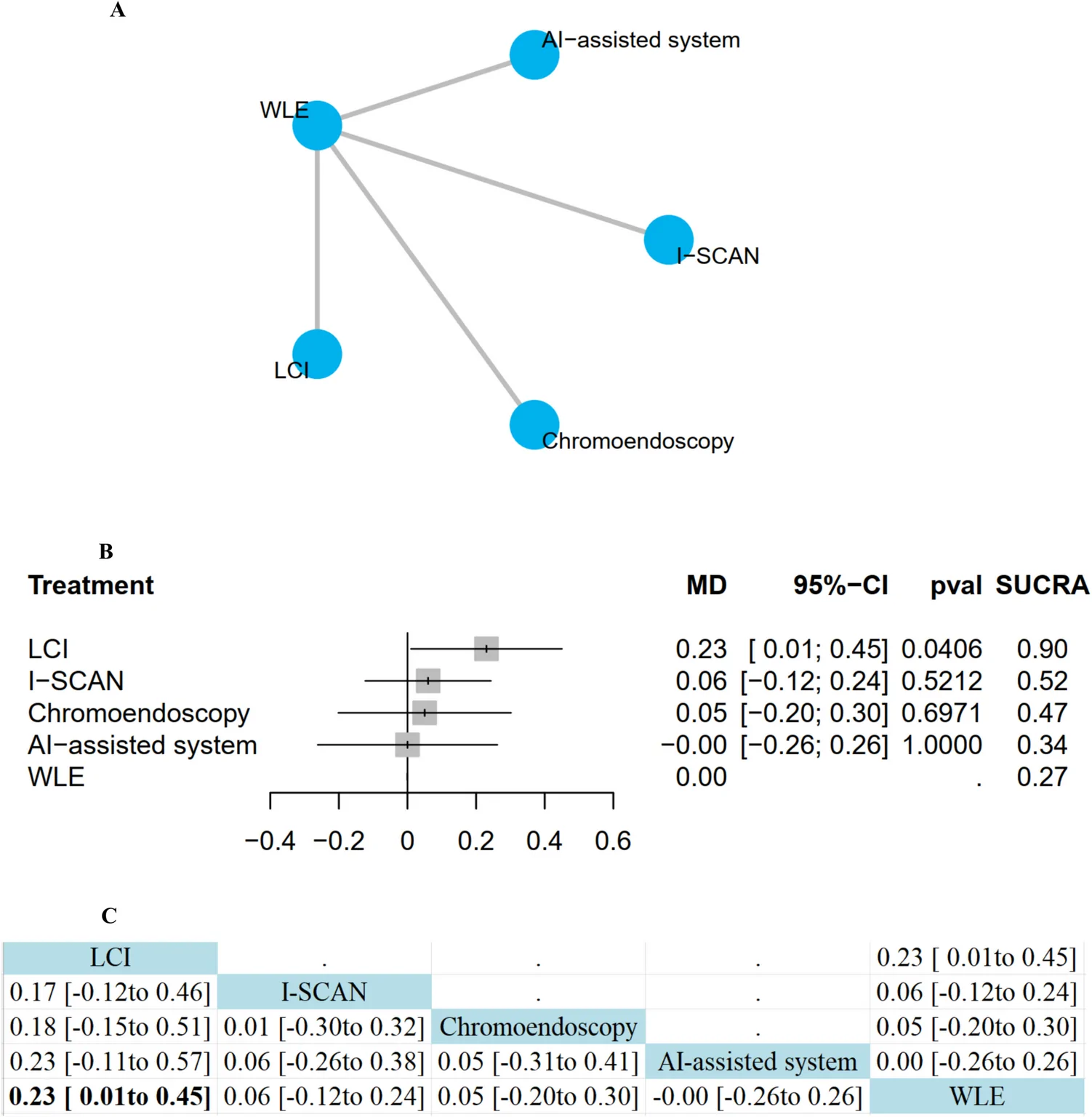
Number of lesions per colonoscopy for neoplastic lesions: **A** network plot, **B** forest plot, and **C** league table

Regarding neoplastic lesions, four studies were included in this analysis. The network plot is presented in Fig. 4A. Only LCI showed a statistically significant difference compared to WLE (MD 0.23, 95% CI [0.01, 0.45], *P* = 0.04, as shown in Fig. 4B). However, no significant difference was observed between any of the arms. According to SUCRA rankings, LCI showed the highest ranking (SUCRA = 90%), while WLE showed the lowest (SUCRA = 27%). The analysis revealed no substantial heterogeneity or inconsistency (Cochran’s *Q* = 3.5, *P* = 0.6739, with *I*^2^ = 0%). As for the size of neoplastic lesions, LCI demonstrated a statistically significant difference compared to WLE in adenomas less than 5 mm (MD 0.20, 95% CI [0.01, 0.39], *P* = 0.04, as shown in Supplementary Fig. 4). The analysis revealed no substantial heterogeneity or inconsistency (Cochran’s *Q* = 0.01, *P* = 0.9264, with *I*^2^ = 0%). However, for adenomas equal to or more than 5 mm, no significant difference existed between the arms and each other (Supplementary Fig. 5). The analysis revealed no substantial heterogeneity or inconsistency (Cochran’s *Q* = 0.48, *P* = 0.4895, with *I*^2^ = 0%).

**Fig. 5:**
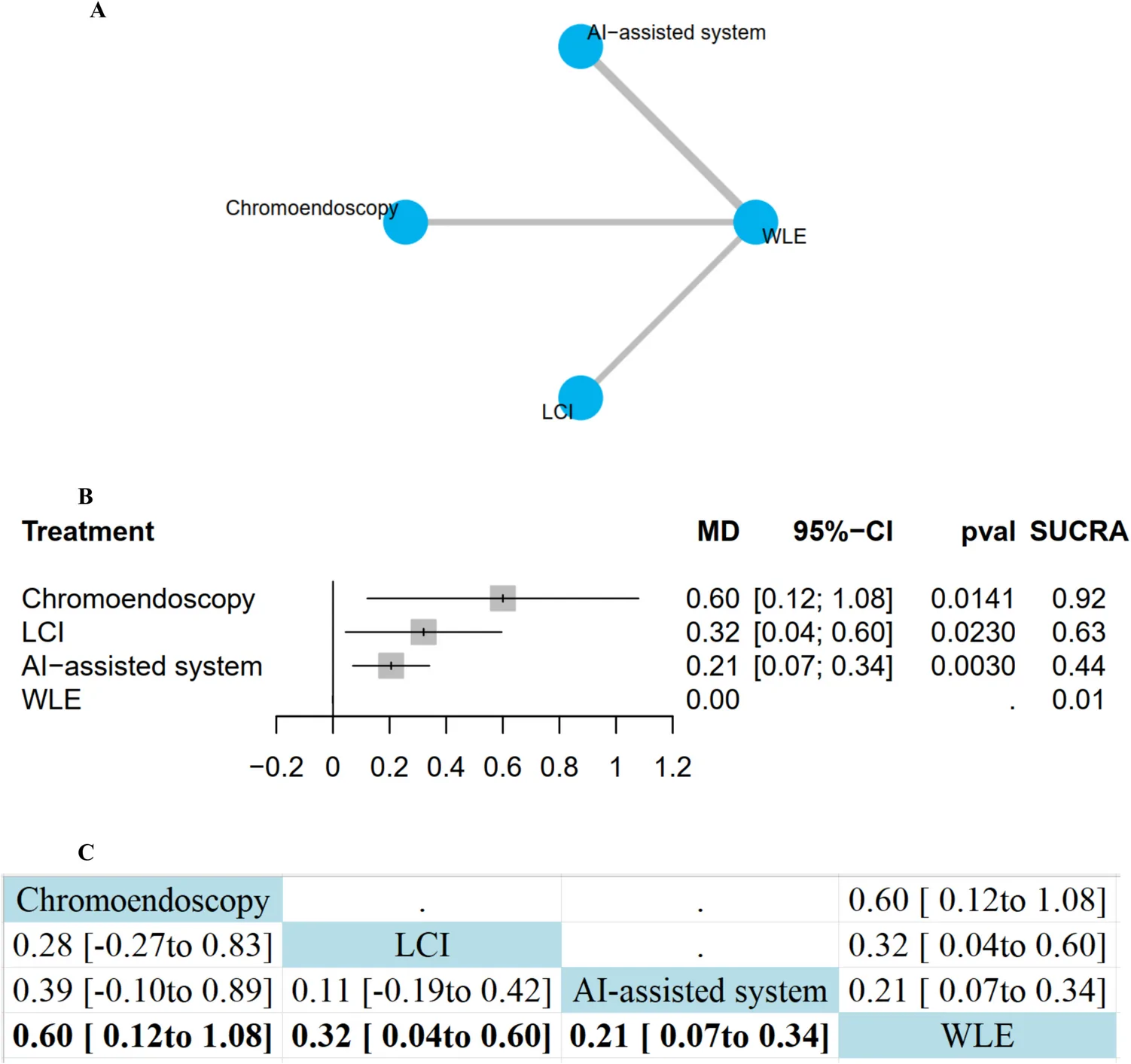
Number of lesions per colonoscopy for non-neoplastic lesions: **A** network plot, **B** forest plot, and **C** league table

Concerning non-neoplastic lesions, four studies were included in this analysis. The corresponding network plot is illustrated in Fig. 5A. Compared to WLE, chromoendoscopy, LCI, and AI-assisted systems showed statistically significant differences (MD 0.60, 95% CI [0.12, 1.08], *P* = 0.01, as shown in Fig. 5B), (MD 0.32, 95% CI [0.04, 0.60], *P* = 0.02, as shown in Fig. 5B), and (MD 0.21, 95% CI [0.07, 0.34], *P* = 0.003, as shown in Fig. 5B), respectively. According to SUCRA rankings, chromoendoscopy achieved the highest ranking (SUCRA = 92%), followed by LCI (SUCRA = 63%) and AI-assisted systems (SUCRA = 44%). No significant difference existed between these modalities and each other. The analysis revealed no substantial heterogeneity or inconsistency (Cochran’s *Q* = 0.33, *P* = 0.5633, with *I*^2^ = 0%). Regarding hyperplastic lesions, chromoendoscopy showed a statistically significant difference compared to WLE (MD 0.48, 95% CI [0.07, 0.89], *P* = 0.02) and achieved the highest SUCRA ranking (SUCRA = 94%), while NBI achieved the lowest (SUCRA = 31%), as shown in Supplementary Fig. 6. The analysis revealed substantial heterogeneity (Cochran’s *Q* = 2.82, *P* = 0.0933, with *I*^2^ = 64.5%). For sessile lesions, no significant difference existed between any of the arms (Supplementary Fig. 7). The analysis revealed no substantial heterogeneity or inconsistency (Cochran’s *Q* = 3.2, *P* = 0.8254, with *I*^2^ = 0%).

#### Secondary outcomes

##### Total procedure time

Comparison of total procedure time across different endoscopic modalities revealed that none of the alternatives significantly reduced procedure time compared to WLE. However, when chromoendoscopy was used as the reference, WLE (MD − 8.71, 95% CI − 10.72 to − 6.70), AI-assisted systems (MD − 8.52, 95% CI − 10.78 to − 6.27), and LCI (MD − 7.04, 95% CI − 9.95 to − 4.13) demonstrated statistically significant reductions in procedure time, as shown in Supplementary Fig. 8. Based on SUCRA rankings, WLE was the most time-efficient modality (SUCRA = 86%), while chromoendoscopy was the most time-consuming (SUCRA = 0%). The analysis revealed no substantial heterogeneity or inconsistency (Cochran’s *Q* = 0.75, *P* = 0.6861, with *I*^2^ = 0%).

##### Withdrawal time

Like total procedure time, none of the different modalities significantly reduced withdrawal time compared to WLE. However, compared to chromoendoscopy, WLE (MD − 7.14, 95% CI − 8.60 to − 5.68), LCI (MD − 6.14, 95% CI − 8.82 to − 3.47), AI-assisted systems (MD − 6.20, 95% CI − 8.13 to − 4.27), and NBI (MD − 6.04, 95% CI − 8.37 to − 3.71) exhibited statistically significant reductions in withdrawal time, as shown in Supplementary Fig. 9. According to SUCRA rankings, WLE ranked first (89%), while chromoendoscopy ranked last (0%). The analysis revealed substantial heterogeneity (Cochran’s *Q* = 15.82, *P* = 0.0033, with *I*^2^ = 74.7%).

### Credibility assessment

The overall confidence in the evidence was judged as low for all comparisons in both neoplastic and non-neoplastic lesion detection outcomes. This was primarily driven by major concerns regarding imprecision, as many credible intervals were wide and frequently crossed clinically meaningful thresholds. Within-study bias also contributed to downgrading, with most studies showing “some concerns” due to methodological limitations. In contrast, indirectness, heterogeneity, and incoherence were not major issues in this network, as the evidence was generally applicable, findings were consistent across studies, and agreement was observed between direct and indirect comparisons. Although the network included a variety of comparisons, the number of studies contributing to most estimates was limited, which restricted the evaluation of publication bias. However, the risk of reporting bias was consistently rated as low, with no evidence of selective outcome reporting identified across the included studies. Table 2 demonstrates the certainty of evidence for the neoplastic lesions detection rate outcome. The certainty of evidence for the non-neoplastic lesions detection rate outcome is represented in Supplementary Table 3.

**Table 2:** Certainty of evidence for the outcome of neoplastic lesions detection rate

Comparison	Number of studies	Within-study bias	Reporting bias	Indirectness	Imprecision	Heterogeneity	Incoherence	Confidence rating
AI-assisted system: WLE	3	Some concerns	Low risk	No concerns	Major concerns	No concerns	No concerns	Low
Chromoendoscopy: NBI	1	Some concerns	Low risk	No concerns	Major concerns	No concerns	No concerns	Low
Chromoendoscopy: WLE	4	Some concerns	Low risk	No concerns	Major concerns	No concerns	No concerns	Low
I-SCAN: WLE	1	Some concerns	Low risk	No concerns	Major concerns	No concerns	No concerns	Low
LCI: WLE	1	Some concerns	Low risk	No concerns	No concerns	Major concerns	No concerns	Low
NBI: WLE	1	Some concerns	Low risk	No concerns	Major concerns	No concerns	No concerns	Low
AI-assisted system: chromoendoscopy	0	Some concerns	Low risk	No concerns	Major concerns	No concerns	No concerns	Low
AI-assisted system: I-SCAN	0	Some concerns	Low risk	No concerns	Major concerns	No concerns	No concerns	Low
AI-assisted system: LCI	0	Some concerns	Low risk	No concerns	No concerns	Major concerns	No concerns	Low
AI-assisted system: NBI	0	Some concerns	Low risk	No concerns	Major concerns	No concerns	No concerns	Low
Chromoendoscopy: I-SCAN	0	Some concerns	Low risk	No concerns	Major concerns	No concerns	No concerns	Low
Chromoendoscopy: LCI	0	Some concerns	Low risk	No concerns	Major concerns	No concerns	No concerns	Low
I-SCAN: LCI	0	Some concerns	Low risk	No concerns	Major concerns	No concerns	No concerns	Low
I-SCAN: NBI	0	Some concerns	Low risk	No concerns	Major concerns	No concerns	No concerns	Low
LCI: NBI	0	Some concerns	Low risk	No concerns	Major concerns	No concerns	No concerns	Low

WLE, white-light endoscopy; NBI, narrow band imaging; LCI, linked color imaging; AI, artificial intelligence

## Discussion

To our knowledge, this was the first systematic review and network meta-analysis that aimed to evaluate and compare the effectiveness of various colonoscopic imaging techniques, including WLE, chromoendoscopy, virtual chromoendoscopy (NBI, LCI, I-SCAN), and AI-assisted colonoscopy, in detecting neoplastic and non-neoplastic lesions in adult patients with Lynch syndrome. The main findings were as follows: LCI and chromoendoscopy provided a better statistically significant difference in the detection of neoplastic lesions compared to conventional WLE, which means that they can better reveal small mucosal changes. This is especially important in the case of Lynch syndrome, where the ability to detect small adenomas early on can make a big difference in patient outcomes [37, 38]. Furthermore, LCI identified more neoplastic lesions per colonoscopy, which makes it a better choice for high-risk populations [39].

Non-neoplastic lesion detection proved most effective with chromoendoscopy, which produced better results than WLE and received the highest ranking in terms of lesion detection. The practical application of dye-based techniques remains important for detecting flat or indistinct lesions, but their use is restricted by the longer procedure and withdrawal times [40]. The diagnostic value of AI-assisted systems and virtual modalities, including I-SCAN and NBI, was observed, but these systems failed to surpass LCI and chromoendoscopy in any of the evaluated outcomes.

From an efficiency standpoint, WLE maintained its position as the quickest method for both total procedure and withdrawal times, while chromoendoscopy required the longest duration. The results demonstrate that LCI provides an optimal balance between diagnostic performance and procedural duration because it enhances detection rates at a reasonable increase in time [41–44].

The results demonstrated that LCI can be used as a practical and effective enhancement to standard colonoscopy for detecting neoplastic lesions in patients with Lynch syndrome. Technically, this superior detection may be attributed to LCI’s digital color enhancement, which improves image contrast to reveal subtle differences between normal and abnormal mucosal and vascular patterns. When it comes to identifying non-neoplastic lesions, such as flat or indistinct lesions, chromoendoscopy has shown outstanding sensitivity. Longer procedure and withdrawal times, however, restrict its practical use. While chromoendoscopy raises detection rates, a study by Houwen et al. (2021) suggests its selective use when the highest sensitivity is needed since the prolonged procedural time may limit its routine use [17]. The importance of AI-assisted procedures in colonoscopy is changing. The research conducted so far indicates that AI technologies help improve detection rates but have not been able to exceed improvements brought by LCI and chromoendoscopy. More work is needed for these devices’ technology before they could supersede standard practices. To the best of our knowledge, this is the first and most comprehensive systematic review and network meta-analysis evaluating the effectiveness of various colonoscopic imaging techniques in Lynch syndrome patients. Our analysis included studies with large sample sizes and participants with different demographic and clinical characteristics, making the results more robust. Heterogeneity across studies was generally low, with most *I*^2^ values at or near 0%, reflecting a high degree of consistency in reported outcomes. While the network included both RCTs and a single cohort study, the latter was assessed separately using the NOS tool, and its contribution was transparently incorporated into the CINeMA confidence grading.

Despite the strengths of this study, several limitations should be acknowledged. The single-center approach, where one endoscopist performed most of the procedures, raises the possibility of operator bias [2, 3, 15]. Second, chromoendoscopy was performed only in the proximal colon in some studies, which may miss some lesions in the distal segments [35]. Third, some studies that excluded small hyperplastic rectosigmoid polyps may have underestimated LCI’s diagnostic performance. Fourth, the limited follow-up period restricted the assessment of long-term outcomes like cancer incidence. Fifth, due to the limited number of studies per outcome, we did not perform funnel plot analyses to assess publication bias, as such plots are unreliable and difficult to interpret with fewer than 10 studies. Lastly, differences in the skill level of the participating endoscopists may have resulted in additional operator bias across included studies [1–4, 7, 15, 17, 35, 36]. These results have important clinical significance. The European Society of Gastrointestinal Endoscopy (ESGE) and the National Comprehensive Cancer Network (NCCN) suggest routine colonoscopy surveillance every 1–2 years for patients with Lynch syndrome, but do not recommend specific imaging techniques [45]. Our results demonstrate that these policies can be changed. Specifically, LCI appears to augment standard colonoscopy due to its better neoplastic detection versus the added time required for the procedure. Thus, LCI is advantageous in high-volume medical facilities where it is important to keep things running smoothly. Although chromoendoscopy has increased the detection rate of non-neoplastic lesions, its adoption into routine practice may be limited due to markedly lengthened procedure times and withdrawal times, increased resource consumption (e.g., dye preparation), and need for operator skill. Thus, it is best reserved for high-risk patients or centers able to accommodate long exam durations [46, 47].

While AI-assisted colonoscopy is still a developing technology, there is promise in it being used as a software addition to existing endoscopic equipment [48–50]. That said, it does not reliably outperform LCI or chromoendoscopy [50–52], and guideline organizations may be waiting for additional multicenter confirmatory studies before broad endorsement of AI applications in colonoscopic imaging. Because of the differing expenditures, training requirements, and facility needs, endpoints should customize the selected technology based on the patient’s risk level, local knowledge, available support, and system assets. Work is still ongoing for AI-assisted colonoscopy, but it can be integrated into current endoscopic devices as software. Still, it does not outperform LCI or chromoendoscopy consistently; guideline groups might wait for further multicenter validation studies before broad recommendations are issued. Due to the varying costs, training demands, infrastructure needs, and differing risks among patients, local expertise, and healthcare resources available, each center should pick the modality tailored most closely to their situation [49, 53]. Future endeavors should concentrate on large multicenter studies with high standards for endoscope training, bowel preparation, and withdrawal time to resolve the existing lack of evidence in Lynch syndrome surveillance. Follow-up to assess clinically meaningful interval outcomes like interval cancer, colorectal cancer-specific mortality, and advanced age imaging like AI and virtual chromoendoscopy should be evaluated and developed for assessment of the entire colon instead of segmental evaluation. Use of AI to generate benchmarks could reduce assessment biases. Blinded outcome assessments could help mitigate the Hawthorne effect—a form of bias in which individuals tend to change their behavior in response to being observed. In addition, pioneering imaging techniques paired with biomarker surveillance warrant cost-effectiveness scrutiny to ensure practical utility geared towards patients rather than simply surveillance. Collaborating internationally through consortia along with data registries will enable the pooling of information as well as validation of data and findings that form the core needs for bulk-dedicated advanced cancer care technologies in patients with Lynch syndrome.

## Conclusion

This network meta-analysis demonstrated that out of all imaging modalities, LCI and chromoendoscopy are the most effective for detecting neoplastic lesions in Lynch syndrome patients. Non-neoplastic lesion detection proved most effective with chromoendoscopy, but it has the drawback of longer withdrawal and overall procedure times. AI-assisted techniques and virtual chromoendoscopy do assist in lesion detection when compared with WLE, but their performance still does not reach that of LCI or chromoendoscopy. Advanced imaging tools should be used selectively depending on the needs and available resources, as these findings suggest. Future research should focus on large-scale, multicenter trials to validate the efficacy of AI and virtual colonoscopic systems and to explore the evolving role of these modalities in comprehensive colonoscopic surveillance.

## Supplementary Information

Below is the link to the electronic supplementary material.

Supplementary file1 (DOCX 3874 KB)

## Data Availability

All data generated or analyzed during this study are included in this published article [and its supplementary information files].

## References

[1] Ortiz O, Daca-Alvarez M, Rivero-Sanchez L, Gimeno-Garcia AZ, Carrillo-Palau M, Alvarez V et al (2024) An artificial intelligence-assisted system versus white light endoscopy alone for adenoma detection in individuals with Lynch syndrome (TIMELY): an international, multicentre, randomised controlled trial. Lancet Gastroenterol Hepatol 9:802–810 10.1016/S2468-1253(24)00187-0 39033774

[2] Montale A, Buttitta F, Pierantoni C, Ferrari C, Cameletti M, Colussi D et al (2022) Chromoendoscopy is not superior to white light endoscopy in improving adenoma detection in Lynch syndrome cohort undergoing surveillance with high-resolution colonoscopy: a real-world evidence study. Dig Dis 40:517–525 10.1159/000518840 34515093

[3] Hüneburg R, Bucksch K, Schmeißer F, Heling D, Marwitz T, Aretz S et al (2023) Real-time use of artificial intelligence (CADEYE) in colorectal cancer surveillance of patients with Lynch syndrome-a randomized controlled pilot trial (CADLY). United European Gastroenterol J 11:60–68 10.1002/ueg2.12354 PMC9892476 36571259

[4] Hüneburg R, Lammert F, Rabe C, Rahner N, Kahl P, Büttner R et al (2009) Chromocolonoscopy detects more adenomas than white light colonoscopy or narrow band imaging colonoscopy in hereditary nonpolyposis colorectal cancer screening. Endoscopy 41:316–322 10.1055/s-0028-1119628 19340735

[5] Tanakaya K (2019) Current clinical topics of Lynch syndrome. Int J Clin Oncol 24:1013–1019 10.1007/s10147-018-1282-7 29744602

[6] Haraldsdottir S, Rafnar T, Frankel WL, Einarsdottir S, Sigurdsson A, Hampel H et al (2017) Comprehensive population-wide analysis of Lynch syndrome in Iceland reveals founder mutations in *MSH6* and *PMS2*. Nat Commun 8:14755 10.1038/ncomms14755 PMC5418568 28466842

[7] Rivero-Sánchez L, Arnau-Collell C, Herrero J, Remedios D, Cubiella J, García-Cougil M et al (2020) White-light endoscopy is adequate for Lynch syndrome surveillance in a randomized and noninferiority study. Gastroenterology 158:895-904.e1 10.1053/j.gastro.2019.09.003 31520613

[8] Pussila M, Laiho A, Törönen P, Björkbacka P, Nykänen S, Pylvänäinen K et al (2024) Mitotic abnormalities precede microsatellite instability in Lynch syndrome-associated colorectal tumourigenesis. EBioMedicine 103:105111 10.1016/j.ebiom.2024.105111 PMC11002576 38583260

[9] Devall M, Ali MW, Eaton S, Weisenberger DJ, Reilley MJ, Powell SM et al (2023) Multi-omic analysis in normal colon organoids highlights MSH4 as a novel marker of defective mismatch repair in Lynch syndrome and microsatellite instability. Cancer Med 12:13551–13572 10.1002/cam4.6048 PMC10315803 37162286

[10] Taieb J, Svrcek M, Cohen R, Basile D, Tougeron D, Phelip J-M (2022) Deficient mismatch repair/microsatellite unstable colorectal cancer: diagnosis, prognosis and treatment. Eur J Cancer 175:136–157 10.1016/j.ejca.2022.07.020 36115290

[11] Moreira L, Balaguer F, Lindor N, de la Chapelle A, Hampel H, Aaltonen LA et al (2012) Identification of Lynch syndrome among patients with colorectal cancer. JAMA 308:1555–1565 10.1001/jama.2012.13088 PMC3873721 23073952

[12] Lynch HT, de la Chapelle A (2003) Hereditary colorectal cancer. N Engl J Med 348:919–932 10.1056/NEJMra012242 12621137

[13] Vasen HFA, Boland CR (2005) Progress in genetic testing, classification, and identification of Lynch syndrome. JAMA 293:2028–2030 10.1001/jama.293.16.2028 15855438

[14] Aaltonen LA, Salovaara R, Kristo P, Canzian F, Hemminki A, Peltomäki P et al (1998) Incidence of hereditary nonpolyposis colorectal cancer and the feasibility of molecular screening for the disease. N Engl J Med 338:1481–1487 10.1056/NEJM199805213382101 9593786

[15] Bisschops R, Tejpar S, Willekens H, De Hertogh G, Van Cutsem E (2017) Virtual chromoendoscopy (I-SCAN) detects more polyps in patients with Lynch syndrome: a randomized controlled crossover trial. Endoscopy 49:342–350 10.1055/s-0042-121005 28107763

[16] Koornstra JJ, Mourits MJ, Sijmons RH, Leliveld AM, Hollema H, Kleibeuker JH (2009) Management of extracolonic tumours in patients with Lynch syndrome. Lancet Oncol 10:400–408 10.1016/S1470-2045(09)70041-5 19341971

[17] Houwen BBSL, Hazewinkel Y, Pellisé M, Rivero-Sánchez L, Balaguer F, Bisschops R et al (2022) Linked colour imaging for the detection of polyps in patients with Lynch syndrome: a multicentre, parallel randomised controlled trial. Gut 71:553–560 10.1136/gutjnl-2020-323132 PMC8862075 34086597

[18] Zhao S, Wang S, Pan P, Xia T, Chang X, Yang X et al (2019) Magnitude, risk factors, and factors associated with adenoma miss rate of tandem colonoscopy: a systematic review and meta-analysis. Gastroenterology 156:1661-1674.e11 10.1053/j.gastro.2019.01.260 30738046

[19] Bressler B, Paszat LF, Chen Z, Rothwell DM, Vinden C, Rabeneck L (2007) Rates of new or missed colorectal cancers after colonoscopy and their risk factors: a population-based analysis. Gastroenterology 132:96–102 10.1053/j.gastro.2006.10.027 17241863

[20] Hutton B, Salanti G, Caldwell DM, Chaimani A, Schmid CH, Cameron C et al (2015) The PRISMA extension statement for reporting of systematic reviews incorporating network meta-analyses of health care interventions: checklist and explanations. Ann Intern Med 162:777–784 10.7326/M14-2385 26030634

[21] Liberati A, Altman DG, Tetzlaff J, Mulrow C, Gøtzsche PC, Ioannidis JPA et al (2009) The PRISMA statement for reporting systematic reviews and meta-analyses of studies that evaluate health care interventions: explanation and elaboration. J Clin Epidemiol 62:1–34 10.1016/j.jclinepi.2009.06.006 19631507

[22] Bramer WM, Giustini D, de Jonge GB, Holland L, Bekhuis T (2016) De-duplication of database search results for systematic reviews in EndNote. J Med Libr Assoc 104:240–243 10.3163/1536-5050.104.3.014 PMC4915647 27366130

[23] Ouzzani M, Hammady H, Fedorowicz Z, Elmagarmid A (2016) Rayyan-a web and mobile app for systematic reviews. Syst Rev 5:210 10.1186/s13643-016-0384-4 PMC5139140 27919275

[24] Ishtiaq R, Zulfiqar L, Gangwani MK, Aziz M (2023) Adenoma detection rate vs. adenoma per colonoscopy as quality indicators for colon cancer screening. Transl Gastroenterol Hepatol 8:24 10.21037/tgh-22-92 PMC10432231 37601737

[25] Mangas-Sanjuan C, Seoane A, Alvarez-Gonzalez MA, Luè A, Suárez A, Álvarez-García V et al (2022) Factors associated with lesion detection in colonoscopy among different indications. United European Gastroenterol J 10:1008–1019 10.1002/ueg2.12325 PMC9731659 36300971

[26] Haghbin H, Zakirkhodjaev N, Aziz M (2023) Withdrawal time in colonoscopy, past, present, and future, a narrative review. Transl Gastroenterol Hepatol 8:19 10.21037/tgh-23-8 PMC10184034 37197256

[27] Sterne JAC, Savović J, Page MJ, Elbers RG, Blencowe NS, Boutron I et al (2019) RoB 2: a revised tool for assessing risk of bias in randomised trials. BMJ 366:l4898 10.1136/bmj.l4898 31462531

[28] Stang A (2010) Critical evaluation of the Newcastle-ottawa scale for the assessment of the quality of nonrandomized studies in meta-analyses. Eur J Epidemiol 25:603–605 10.1007/s10654-010-9491-z 20652370

[29] R: the R project for statistical computing [Internet]. [cited 2025 Apr 22]. Available from: https://www.r-project.org/

[30] Balduzzi S, Rücker G, Nikolakopoulou A, Papakonstantinou T, Salanti G, Efthimiou O, et al. (2023) netmeta : an *R* package for network meta-analysis using frequentist methods. J Stat Softw. 106

[31] Higgins JPT, Thompson SG, Deeks JJ, Altman DG (2003) Measuring inconsistency in meta-analyses. BMJ 327:557–560 10.1136/bmj.327.7414.557 PMC192859 12958120

[32] Nikolakopoulou A, Higgins JPT, Papakonstantinou T, Chaimani A, Del Giovane C, Egger M et al (2020) CINeMA: an approach for assessing confidence in the results of a network meta-analysis. PLoS Med 17:e1003082 10.1371/journal.pmed.1003082 PMC7122720 32243458

[33] Papakonstantinou T, Nikolakopoulou A, Higgins JPT, Egger M, Salanti G (2020) CINeMA: software for semiautomated assessment of the confidence in the results of network meta-analysis. Campbell Syst Rev 16:e1080 10.1002/cl2.1080 PMC8356302 37131978

[34] Sterne JAC, Sutton AJ, Ioannidis JPA, Terrin N, Jones DR, Lau J et al (2011) Recommendations for examining and interpreting funnel plot asymmetry in meta-analyses of randomised controlled trials. BMJ 343:d4002 10.1136/bmj.d4002 21784880

[35] Haanstra JF, Dekker E, Cats A, Nagengast FM, Hardwick JC, Vanhoutvin SA et al (2019) Effect of chromoendoscopy in the proximal colon on colorectal neoplasia detection in Lynch syndrome: a multicenter randomized controlled trial. Gastrointest Endosc 90:624–632 10.1016/j.gie.2019.04.227 31028782

[36] Wei MT, Shankar U, Parvin R, Abbas SH, Chaudhary S, Friedlander Y et al (2023) Evaluation of computer-aided detection during colonoscopy in the community (AI-SEE): a multicenter randomized clinical trial. Am J Gastroenterol 118:1841–1847 10.14309/ajg.0000000000002239 36892545

[37] Castillo-Iturra J, Sánchez A, Balaguer F (2024) Colonoscopic surveillance in Lynch syndrome: guidelines in perspective. Fam Cancer 23:459–468 10.1007/s10689-024-00414-y PMC11512898 39066849

[38] Sánchez A, Roos VH, Navarro M, Pineda M, Caballol B, Moreno L et al (2022) Quality of colonoscopy is associated with adenoma detection and postcolonoscopy colorectal cancer prevention in Lynch syndrome. Clin Gastroenterol Hepatol 20:611-621.e9 10.1016/j.cgh.2020.11.002 33157315

[39] Suzuki S, Aniwan S, Chiu H-M, Laohavichitra K, Chirapongsathorn S, Yamamura T et al (2023) Linked-color imaging detects more colorectal adenoma and serrated lesions: an international randomized controlled trial. Clin Gastroenterol Hepatol 21:1493-1502.e4 10.1016/j.cgh.2022.10.021 36328306

[40] Pal P, Singh AP, Kanuri ND, Banerjee R (2022) Electronic chromo-endoscopy: technical details and a clinical perspective. Transl Gastroenterol Hepatol 7:6 10.21037/tgh-19-373 PMC8826039 35243115

[41] Ikematsu H, Murano T, Shinmura K (2022) Detection of colorectal lesions during colonoscopy. DEN Open 2:e68 10.1002/deo2.68 PMC8828173 35310752

[42] Min M, Deng P, Zhang W, Sun X, Liu Y, Nong B (2017) Comparison of linked color imaging and white-light colonoscopy for detection of colorectal polyps: a multicenter, randomized, crossover trial. Gastrointest Endosc 86:724–730 10.1016/j.gie.2017.02.035 28286095

[43] Lovász BD, Szalai M, Oczella L, Finta Á, Dubravcsik Z, Madácsy L (2020) Improved adenoma detection with linked color imaging technology compared to white-light colonoscopy. Scand J Gastroenterol 55:877–883 10.1080/00365521.2020.1786850 32657195

[44] Sakamoto T, Tomizawa Y, Cho H, Takamaru H, Sekiguchi M, Yamada M et al (2019) Additional value of linked color imaging in colonoscopy: a retrospective study. Endosc Int Open 7:E1448–E1454 10.1055/a-0982-2904 PMC6805211 31673617

[45] van Leerdam ME, Roos VH, van Hooft JE, Balaguer F, Dekker E, Kaminski MF et al (2019) Endoscopic management of Lynch syndrome and of familial risk of colorectal cancer: European society of gastrointestinal endoscopy (ESGE) guideline. Endoscopy 51:1082–1093 10.1055/a-1016-4977 31597170

[46] Feuerstein JD, Rakowsky S, Sattler L, Yadav A, Foromera J, Grossberg L et al (2019) Meta-analysis of dye-based chromoendoscopy compared with standard- and high-definition white-light endoscopy in patients with inflammatory bowel disease at increased risk of colon cancer. Gastrointest Endosc 90:186-195.e1 10.1016/j.gie.2019.04.219 31009609

[47] Kahi CJ (2012) Chromocolonoscopy for colorectal cancer screening: dive into the big blue. J Interv Gastroenterol 2:112–113 10.4161/jig.23729 PMC3655362 23805388

[48] Miyaguchi K, Tsuzuki Y, Hirooka N, Matsumoto H, Ohgo H, Nakamoto H et al (2024) Linked-color imaging with or without artificial intelligence for adenoma detection: a randomized trial. Endoscopy 56:376–383 10.1055/a-2239-8145 PMC11038826 38191000

[49] Taghiakbari M, Mori Y, von Renteln D (2021) Artificial intelligence-assisted colonoscopy: a review of current state of practice and research. World J Gastroenterol 27:8103–8122 10.3748/wjg.v27.i47.8103 PMC8704267 35068857

[50] Shahsavari D, Waqar M, Thoguluva CV (2023) Image enhanced colonoscopy: updates and prospects-a review. Transl Gastroenterol Hepatol 8:26 10.21037/tgh-23-17 PMC10432234 37601740

[51] Arantes V, Campanati RG (2019) Is LCI the best for virtual chromoendoscopy? Endosc Int Open 7:E1455–E1456 10.1055/a-0996-8205 PMC6805209 31682663

[52] Spadaccini M, Menini M, Massimi D, Rizkala T, De Sire R, Alfarone L et al (2025) AI and polyp detection during colonoscopy. Cancers (Basel). 10.3390/cancers17050797 PMC11898786 40075645

[53] Kandiah K, Subramaniam S, Thayalasekaran S, Chedgy FJ, Longcroft-Wheaton G, Fogg C et al (2021) Multicentre randomised controlled trial on virtual chromoendoscopy in the detection of neoplasia during colitis surveillance high-definition colonoscopy (the VIRTUOSO trial). Gut 70:1684–1690 10.1136/gutjnl-2020-320980 PMC8355878 33214162

