# Supplementary material for: Optimizing surveillance in Lynch syndrome: lesion detection and comparative performance of different colonoscopy modalities—a systematic review and network meta-analysis
Source: Int J Colorectal Dis. 2025 Aug 12;40(1):175. doi: 10.1007/s00384-025-04970-2 (PMC12343729; doi:10.1007/s00384-025-04970-2)
Supplement: Supplementary file 1 — Supplementary file1 (DOCX 3874 KB) [file 384_2025_4970_MOESM1_ESM.docx]

| PubMed, WOS, Scopus | (Endoscopy OR colonoscopy OR WLE OR "White-light endoscopy" OR "White-light endoscopy" OR "light endoscopy" OR "standard definition WLE" OR "high definition WLE" OR chromoendoscopy OR CE OR "dye-based chromoendoscopy" OR "virtual chromoendoscopy" OR NBI OR "Narrow band imaging" OR LCI OR "Linked color imaging" OR I-SCAN OR AFE OR "Autofluorescence endoscopy" OR "Artificial intelligence-assisted system" OR "AI-assisted endoscopy" OR "AI-assisted colonoscopy" OR "Artificial intelligence" OR FICE OR "Fuji intelligent chromoendoscopy" OR CADEYE OR "GI Genius" OR EndoBRAIN OR "DEEP Blue AI" OR "Endo-AI" OR "Kiki AI" OR "Veye Colon" OR CADe OR "Computed aided detection systems" OR "Cap-assisted" OR "capsule endoscopy" OR "Virtual endoscopy") AND ("Lynch syndrome" OR "Lynch cancer syndrome" OR "Hereditary nonpolyposis colorectal cancer" OR "HNPCC" OR "Lynch hereditary colorectal cancer syndrome" OR "Muir-Torre syndrome" OR "Turcot syndrome") |
| --- | --- |

**Supplementary Table 1:** Search strategy used in all databases


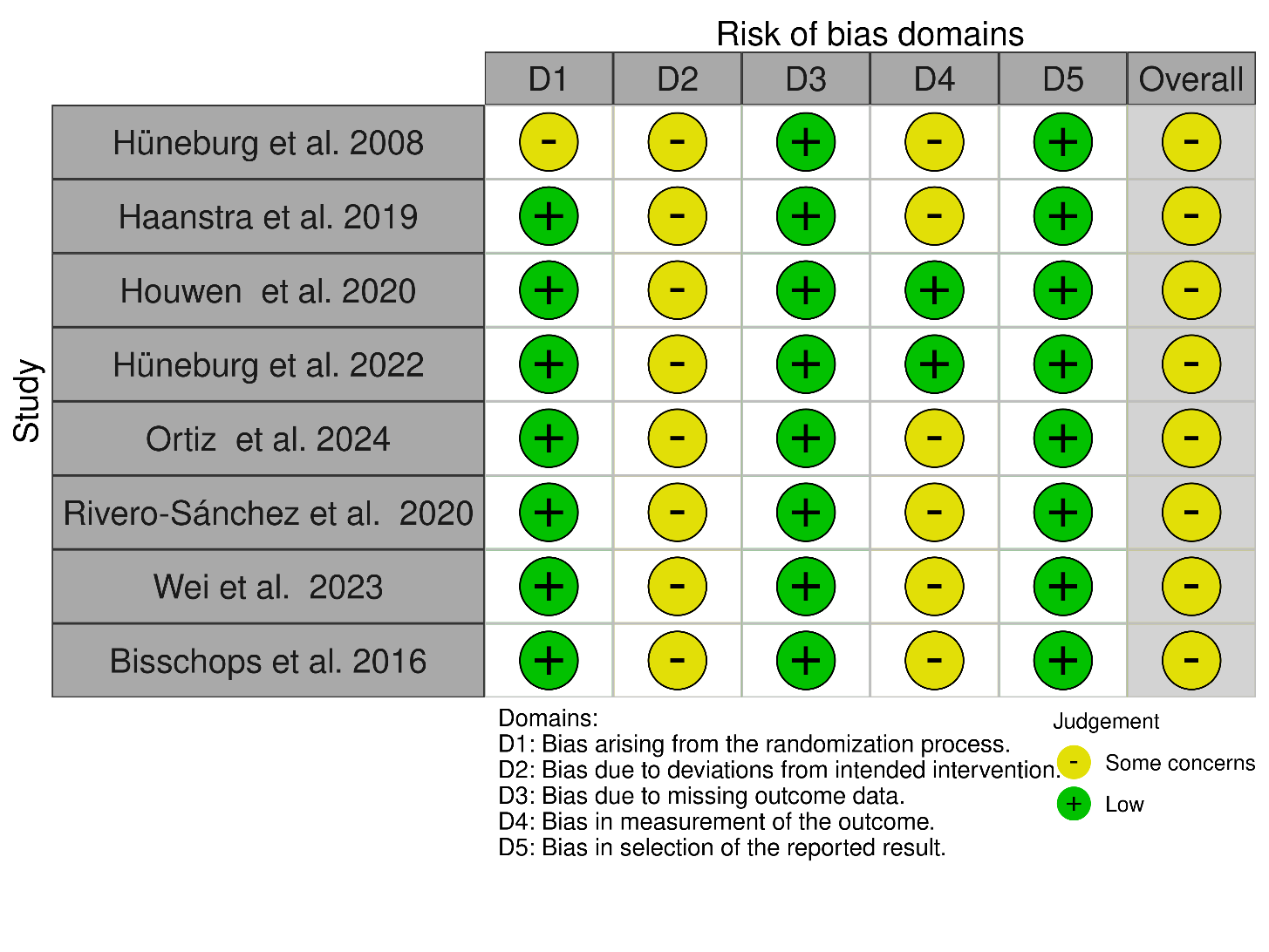

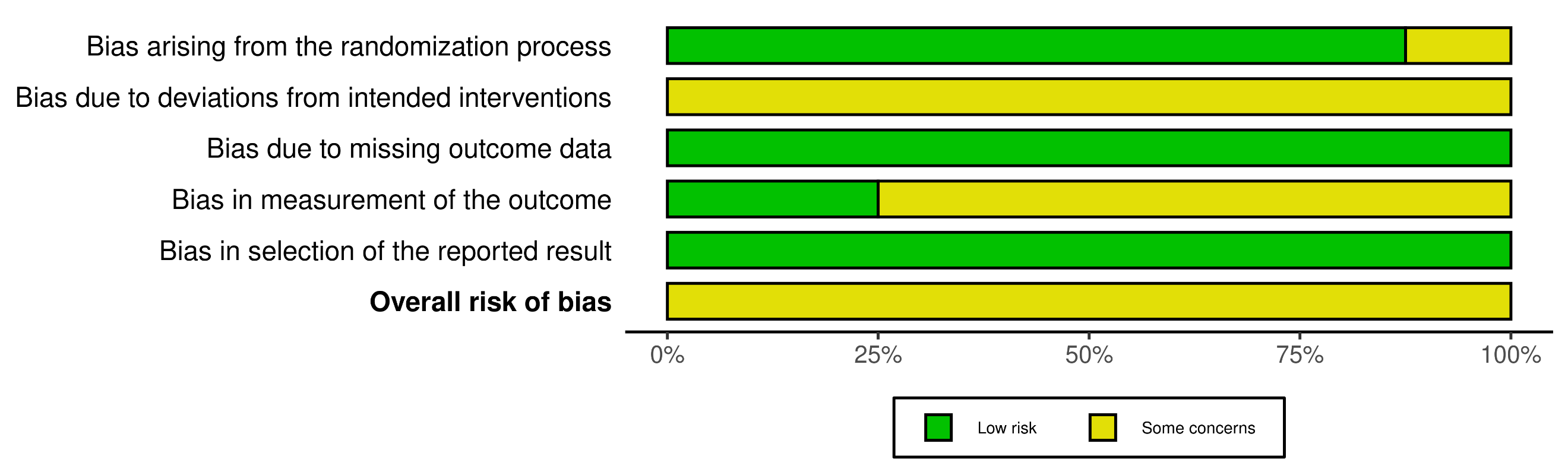


**Supplementary Figure 1:** Risk of Bias assessment for included RCTs using the ROB2 tool

| **Supplementary Table 2:** Newcastle-Ottawa Scale of the included cohort study  **Study number** | **Author and year** | **Selection** | | | | **Comparability** | **Outcome** | | | **Total Score** | **Quality** |
| --- | --- | --- | --- | --- | --- | --- | --- | --- | --- | --- | --- |
|  |  | **Representativeness of the exposed cohort** | **Selection of the non-exposed cohort** | **Ascertainment of exposure** | **Demonstration that outcome of interest was not present at start of study** |  | **Assessment of outcome** | **Follow-up long enough** | **Adequacy of follow up** |  |  |
| **1** | **Montale et al. 2022** | * | * | * | - | * | * | * | * | 7 | Good |


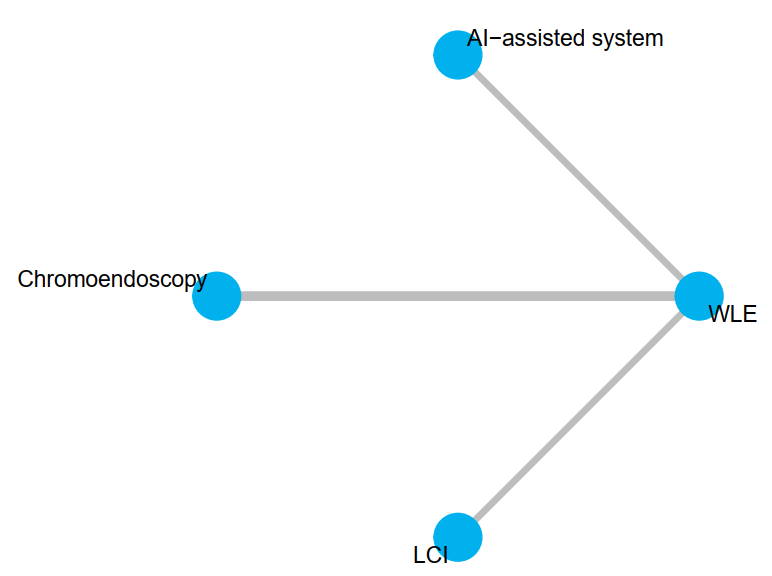


**A**

**B**

**C**


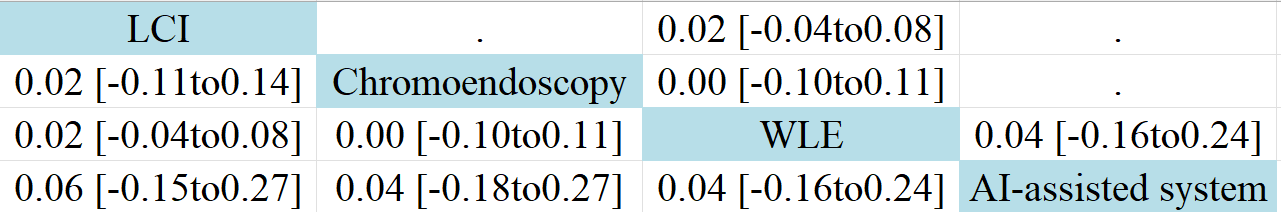


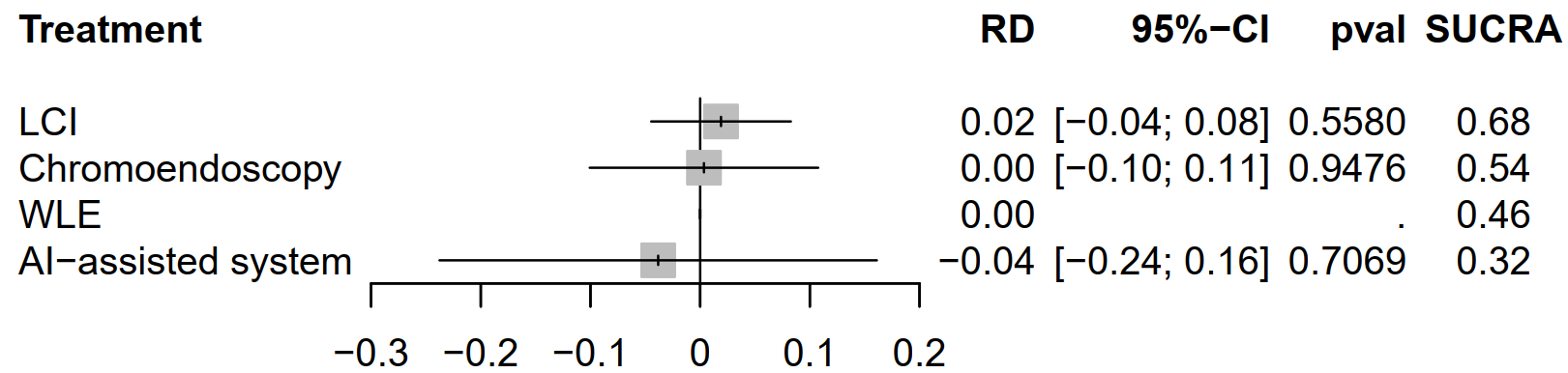


**Supplementary Figure 2:** Lesion detection rate for hyperplastic non-neoplastic lesions **A)** Network plot; **B)** Forest plot; **C)** League table


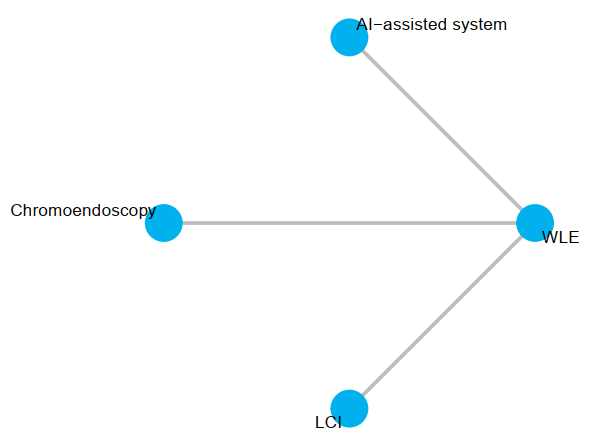


**A**

**B**

**C**


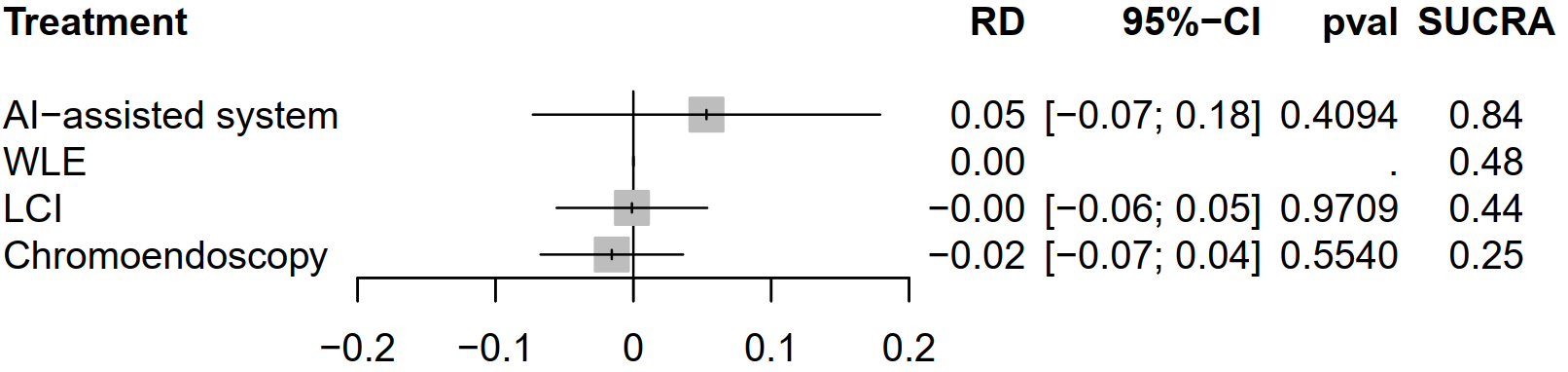

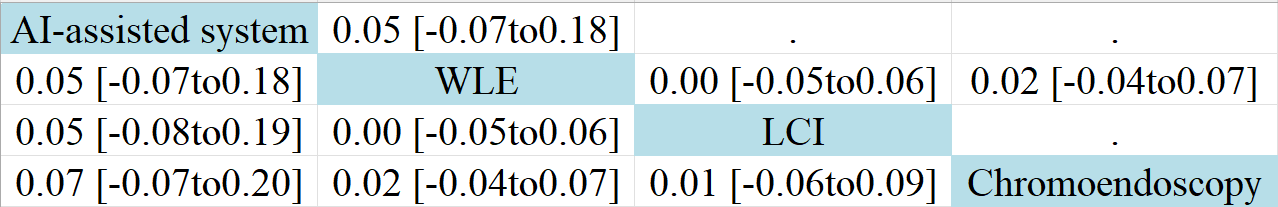


**Supplementary Figure 3:** Lesion detection rate for sessile non-neoplastic lesions **A)** Network plot; **B)** Forest plot; **C)** League table


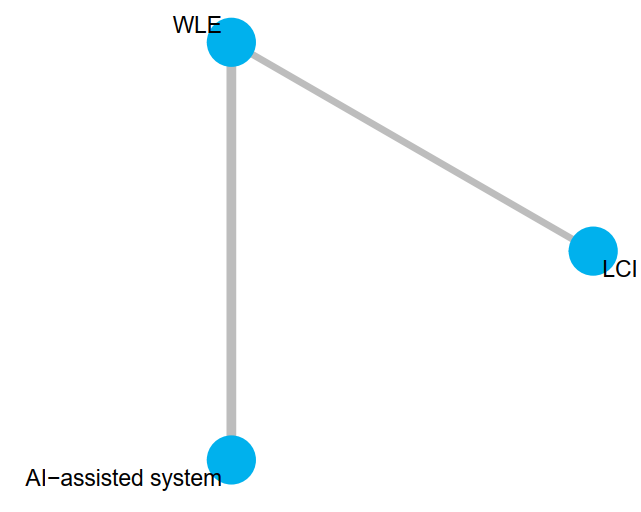

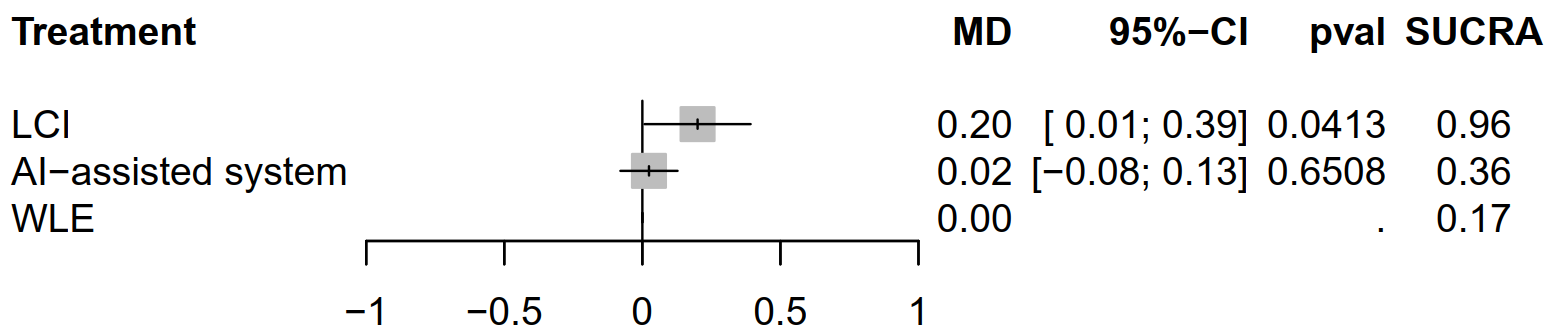

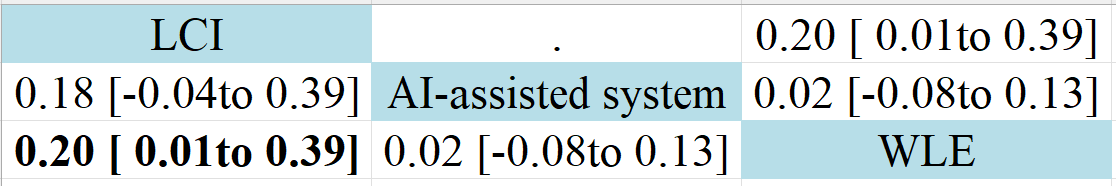


**A**

**B**

**C**

**Supplementary Figure 4:** Number of lesions per colonoscopy for neoplastic lesions less than 5mm **A)** Network plot; **B)** Forest plot; **C)** League table


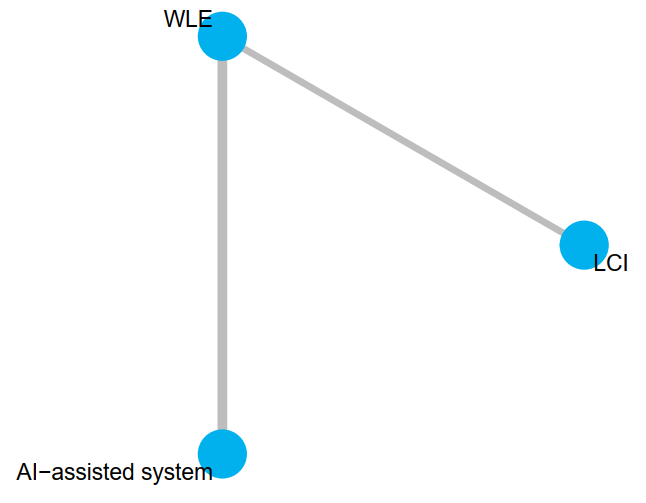

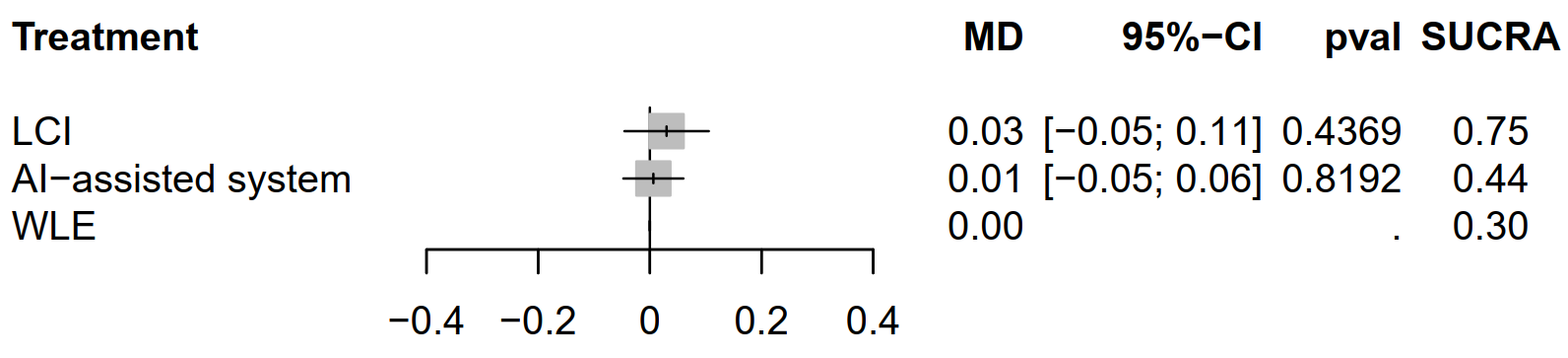

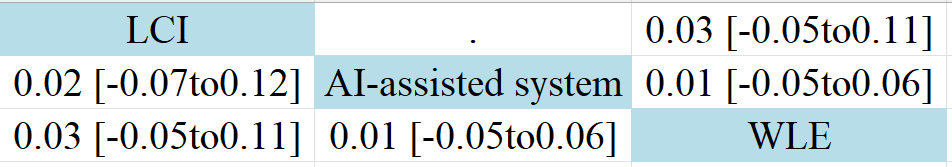


**A**

**B**

**C**

**Supplementary Figure 5:** Number of lesions per colonoscopy for neoplastic lesions equal to or more than 5mm **A)** Network plot; **B)** Forest plot; **C)** League table


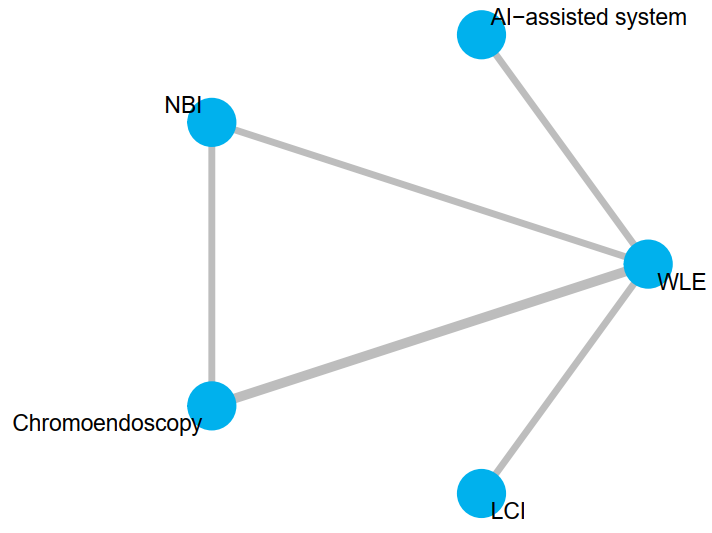

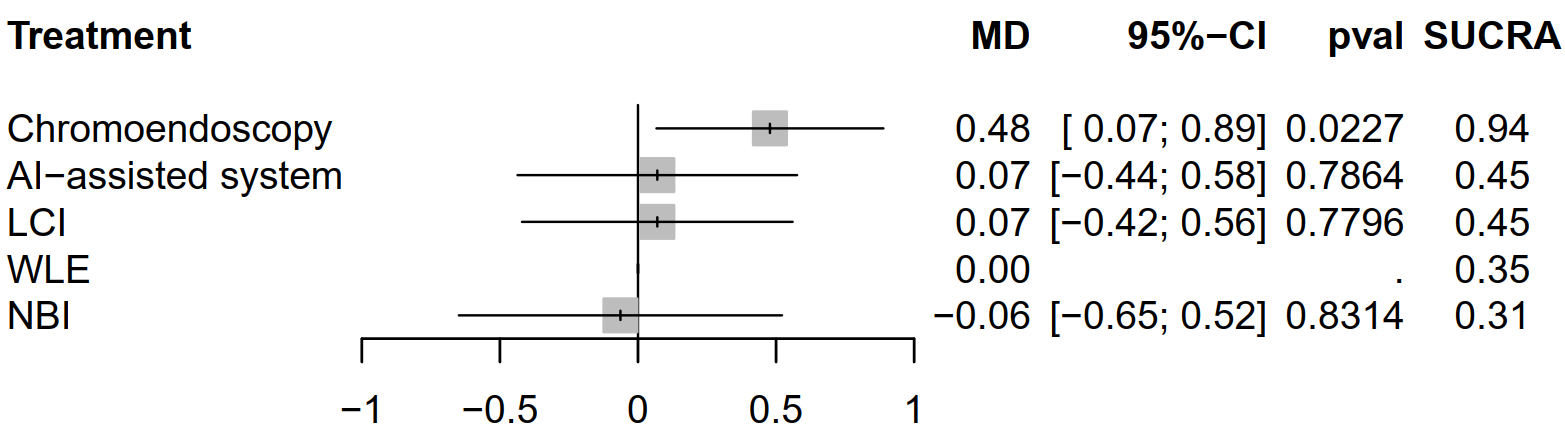

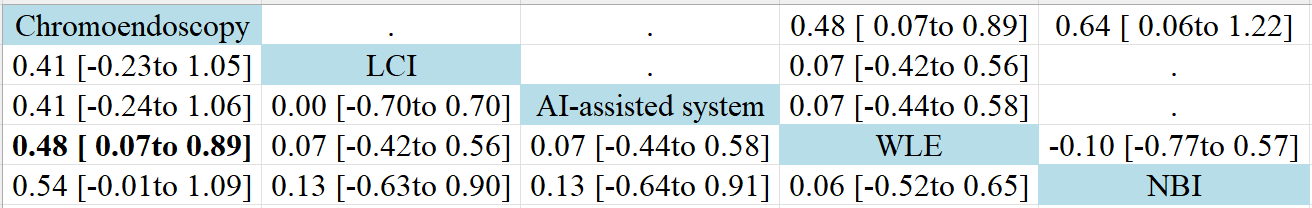


**A**

**B**

**C**

**Supplementary Figure 6:** Number of lesions per colonoscopy for hyperplastic non-neoplastic lesions **A)** Network plot; **B)** Forest plot; **C)** League table


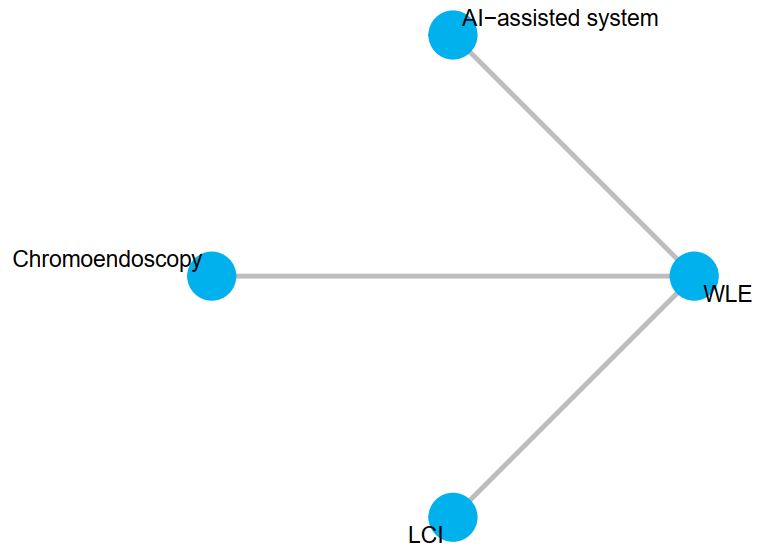

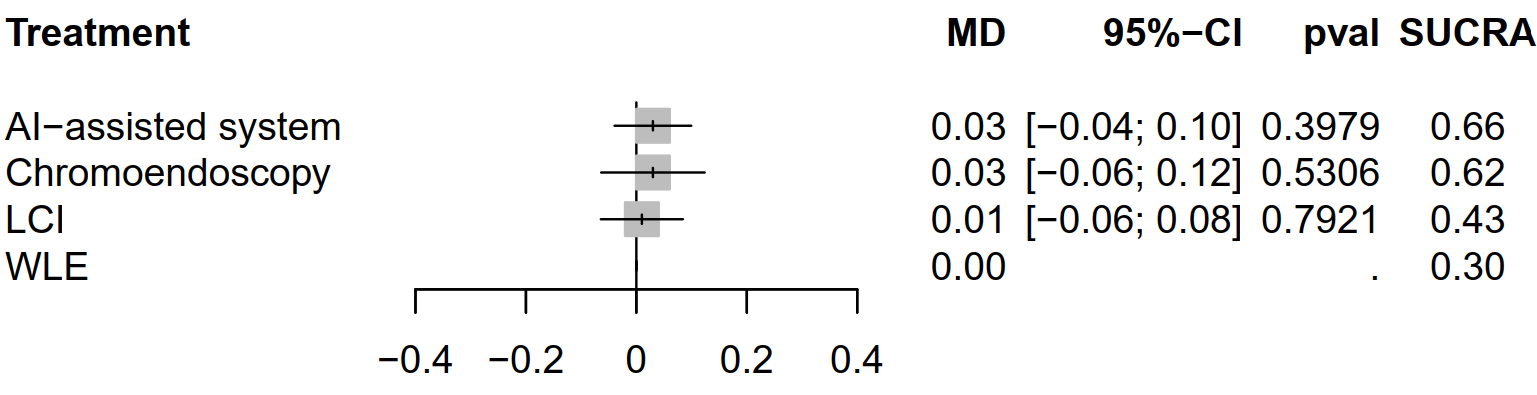

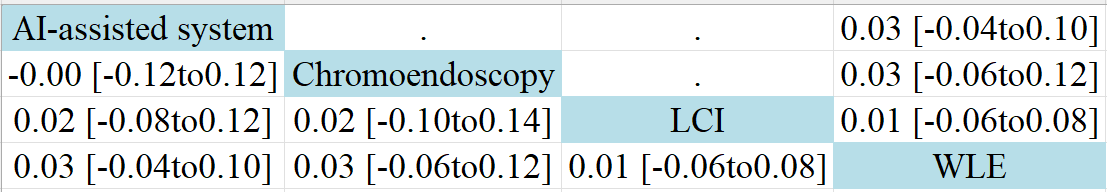


**A**

**B**

**C**

**Supplementary Figure 7:** Number of lesions per colonoscopy for sessile non-neoplastic lesions **A)** Network plot; **B)** Forest plot; **C)** League table


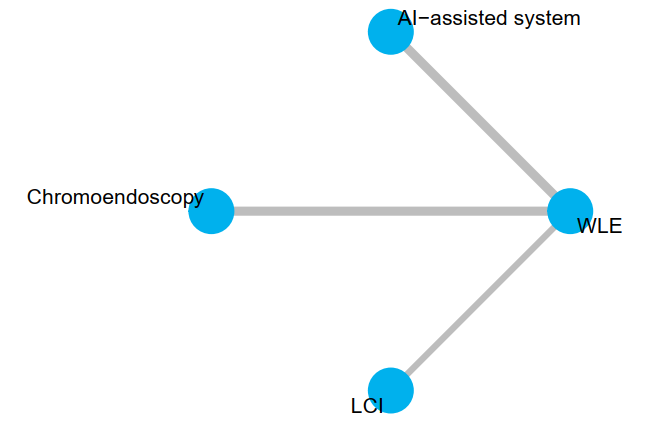

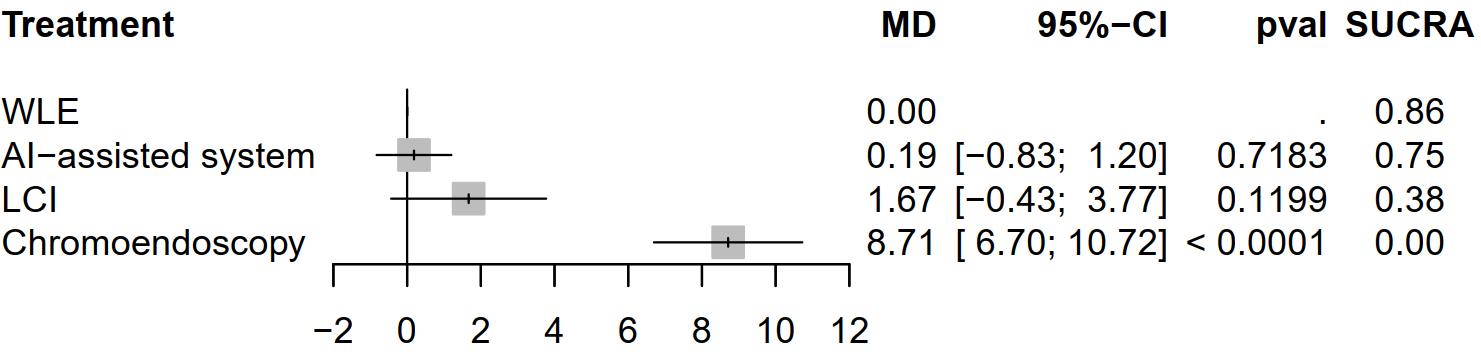

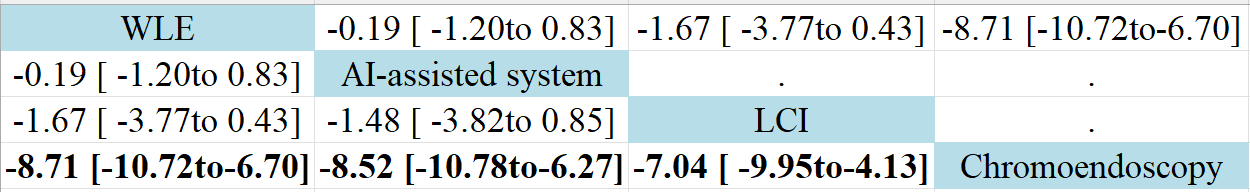


**A**

**B**

**C**

**Supplementary Figure 8:** Total procedure time **A)** Network plot; **B)** Forest plot; **C)** League table


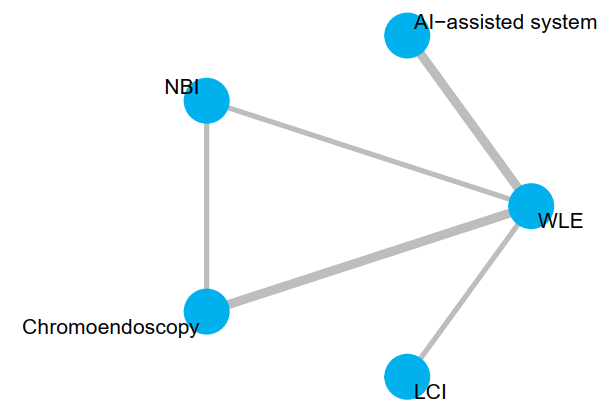

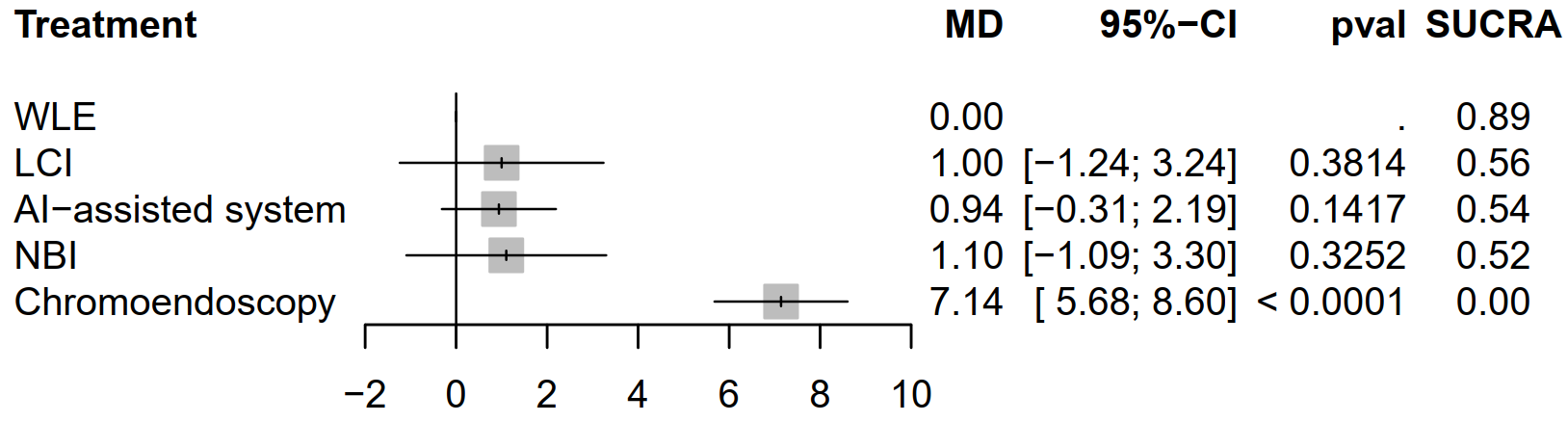

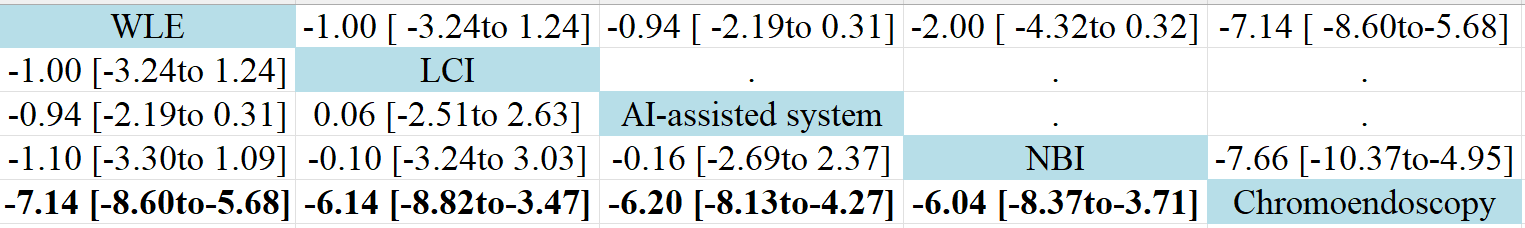


**A**

**B**

**C**

**Supplementary Figure 9:** Withdrawal time **A)** Network plot; **B)** Forest plot; **C)** League table

| **Comparison** | **Number of studies** | **Within-study bias** | **Reporting bias** | **Indirectness** | **Imprecision** | **Heterogeneity** | **Incoherence** | **Confidence rating** |
| --- | --- | --- | --- | --- | --- | --- | --- | --- |
| **AI-assisted system: WLE** | **2** | **Some concerns** | **Low risk** | **No concerns** | **Major concerns** | **No concerns** | **No concerns** | **Low** |
| **Chromoendoscopy: WLE** | **3** | **Some concerns** | **Low risk** | **No concerns** | **No concerns** | **Major concerns** | **No concerns** | **Low** |
| **I-SCAN: WLE** | **1** | **Some concerns** | **Low risk** | **No concerns** | **Major concerns** | **No concerns** | **No concerns** | **Low** |
| **LCI: WLE** | **1** | **Some concerns** | **Low risk** | **No concerns** | **Major concerns** | **No concerns** | **No concerns** | **Low** |
| **AI-assisted system: Chromoendoscopy** | **0** | **Some concerns** | **Low risk** | **No concerns** | **Major concerns** | **No concerns** | **No concerns** | **Low** |
| **AI-assisted system: I-SCAN** | **0** | **Some concerns** | **Low risk** | **No concerns** | **Major concerns** | **No concerns** | **No concerns** | **Low** |
| **AI-assisted system: LCI** | **0** | **Some concerns** | **Low risk** | **No concerns** | **Major concerns** | **No concerns** | **No concerns** | **Low** |
| **Chromoendoscopy: I-SCAN** | **0** | **Some concerns** | **Low risk** | **No concerns** | **Major concerns** | **No concerns** | **No concerns** | **Low** |
| **Chromoendoscopy: LCI** | **0** | **Some concerns** | **Low risk** | **No concerns** | **Major concerns** | **No concerns** | **No concerns** | **Low** |
| **I-SCAN: LCI** | **0** | **Some concerns** | **Low risk** | **No concerns** | **Major concerns** | **No concerns** | **No concerns** | **Low** |

**Supplementary Table 3:** Certainty of evidence for the outcome of non-neoplastic lesions detection rate

| Detection rate for non-neoplastic lesions | | | | |
| --- | --- | --- | --- | --- |
| Study | Study arms | Number of patients with at least one detected lesion | Total number of patients | Detection rate |
| Houwen et al. 2020 | WLE | 62 | 172 | 36.04% |
|  | LCI | 71 | 160 | 44.37% |
| Hüneburg et al. 2022 | WLE | 34 | 46 | 73.91% |
|  | AI-assisted system | 42 | 50 | 84.00% |
| Montale et al. 2022 | WLE | 11 | 29 | 37.93% |
|  | Chromoendoscopy | 6 | 13 | 46.15% |
| Ortiz et al. 2024 | WLE | 107 | 216 | 49.53% |
|  | AI-assisted system | 121 | 214 | 56.54% |
| Rivero-Sánchez et al. 2020 | WLE | 64 | 128 | 50.00% |
|  | Chromoendoscopy | 86 | 128 | 67.18% |
| Bisschops et al. 2016 | WLE | 11 | 31 | 35.48% |
|  | I-SCAN | 11 | 30 | 36.66% |

**Supplementary Table 4:** Studies reported detection rates for non-neoplastic lesions.

**Supplementary Table 5:** Studies reported detection rates for neoplastic lesions.

| Detection rate for neoplastic lesions | | | | |
| --- | --- | --- | --- | --- |
| Study | Study arms | Number of patients with at least one detected lesion | Total number of patients | Detection rate |
| Hüneburg et al. 2008 | WLE | 7 | 47 | 14.89% |
|  | Chromoendoscopy | 9 | 47 | 19.14% |
|  | NBI | 9 | 62 | 14.51% |
|  | Chromoendoscopy | 22 | 62 | 35.48% |
| Haanstra et al. 2019 | WLE | 31 | 116 | 26.72% |
|  | Chromoendoscopy | 35 | 115 | 30.43% |
| Houwen et al. 2020 | WLE | 44 | 172 | 25.58% |
|  | LCI | 58 | 160 | 36.25% |
| Hüneburg et al. 2022 | WLE | 12 | 46 | 26.08% |
|  | AI-assisted system | 18 | 50 | 36.00% |
| Montale et al. 2022 | WLE | 5 | 29 | 17.24% |
|  | Chromoendoscopy | 3 | 13 | 23.07% |
| Ortiz et al. 2024 | WLE | 79 | 216 | 36.57% |
|  | AI-assisted system | 70 | 214 | 32.71% |
| Rivero-Sánchez et al. 2020 | WLE | 36 | 128 | 28.12% |
|  | Chromoendoscopy | 44 | 128 | 34.37% |
| Wei et al. 2023 | WLE | 142 | 382 | 37.17% |
|  | AI-assisted system | 139 | 387 | 35.91% |
| Bisschops et al. 2016 | WLE | 6 | 31 | 19.35% |
|  | I-SCAN | 9 | 30 | 30.00% |
